# Post-GWAS screening of candidate genes for refractive error in mutant zebrafish models

**DOI:** 10.1038/s41598-023-28944-y

**Published:** 2023-02-03

**Authors:** Wim H. Quint, Kirke C. D. Tadema, Nina C. C. J. Kokke, Magda A. Meester-Smoor, Adam C. Miller, Rob Willemsen, Caroline C. W. Klaver, Adriana I. Iglesias

**Affiliations:** 1 Department of Ophthalmology, Erasmus Medical Center, Rotterdam, The Netherlands; 2 Department of Clinical Genetics, Erasmus Medical Center, Rotterdam, The Netherlands; 3 Department of Epidemiology, Erasmus Medical Center, Rotterdam, The Netherlands; 4 Institute of Neuroscience, University of Oregon, Eugene, USA; 5 Department of Ophthalmology, Radboud University Medical Center, Nijmegen, The Netherlands; 6 Institute of Molecular and Clinical Ophthalmology Basel, Basel, Switzerland

**Keywords:** Functional genomics, Disease model, Experimental organisms, Visual system

## Abstract

Genome-wide association studies (GWAS) have dissected numerous genetic factors underlying refractive errors (RE) such as myopia. Despite significant insights into understanding the genetic architecture of RE, few studies have validated and explored the functional role of candidate genes within these loci. To functionally follow-up on GWAS and characterize the potential role of candidate genes on the development of RE, we prioritized nine genes (*TJP2*, *PDE11A*, *SHISA6*, *LAMA2*, *LRRC4C*, *KCNQ5*, *GNB3*, *RBFOX1*, and *GRIA4*) based on biological and statistical evidence; and used CRISPR/cas9 to generate knock-out zebrafish mutants. These mutant fish were screened for abnormalities in axial length by spectral-domain optical coherence tomography and refractive status by eccentric photorefraction at the juvenile (2 months) and adult (4 months) developmental stage. We found a significantly increased axial length and myopic shift in refractive status in three of our studied mutants, indicating a potential involvement of the human orthologs (*LAMA2*, *LRRC4C*, and *KCNQ5*) in myopia development. Further, in-situ hybridization studies showed that all three genes are expressed throughout the zebrafish retina. Our zebrafish models provide evidence of a functional role of these three genes in refractive error development and offer opportunities to elucidate pathways driving the retina-to-sclera signaling cascade that leads to myopia.

## Introduction

Refractive errors (RE) are the most frequent eye disorders which arise when the optics of the eye are not matched to the biometry of the eye. Especially myopia, or nearsightedness, has shown a rapid increase in prevalence over the last decades. This RE is linked to eye diseases such as myopic macular degeneration, glaucoma, cataract, and retinal detachment and is thereby becoming an increasingly common cause of blindness^1–3^.

In the last decade, genome-wide association studies (GWASs) have focused on dissecting the genetic factors underlying RE. In 2018, Tedja et al., identified 161 loci associated with RE in a large (160,420 participants) GWASs meta-analysis^4^. This list was extended to 449 loci in 2020 by a GWASs meta-analysis by Hysi et al., encompassing 542,934 participants of mixed ancestries from various studies including UK Biobank, the GERA, 23andMe and the CREAM consortium^5^. Candidate genes potentially involved in pathways driving the retina-to-sclera signaling cascade underlying RE were annotated to the identified loci, and disease mechanisms such as (cat)ion transport, neurotransmission, neuronal development, cell–cell adhesion, and extracellular matrix remodeling were suggested^4–6^. However, studies exploring the functional roles of these candidates and thereby validating GWAS findings are still scarce. The goal of this functional study is to evaluate the potential of candidate RE genes in controlling refractive error using zebrafish (Danio rerio) as a model. We have previously demonstrated that the zebrafish is an efficient model to study RE^7,8^, which offers a significant improvement in time and costs compared to the more traditionally used avian or mammalian animal models. Using zebrafish, we have previously shown that depletion of *GJD2* (located in one of the genome-wide top-associated signals) leads to the development of RE^8^.

In this study, we investigated nine additional GWAS candidate genes (*TJP2*, *PDE11A*, *SHISA6*, *LAMA2*, *LRRC4C*, *KCNQ5*, *GNB3*, *RBFOX1*, and *GRIA4*) which were prioritized based on statistical (e.g., effect size of associated single nucleotide polymorphisms (SNPs) in GWAS studies) and biological (e.g., ocular function and expression) evidence for their functional role in RE development. Here, we show that three of these candidate genes have a potential role in the determination of eye size.

## Results

### Gene prioritization

**Figure 1:**
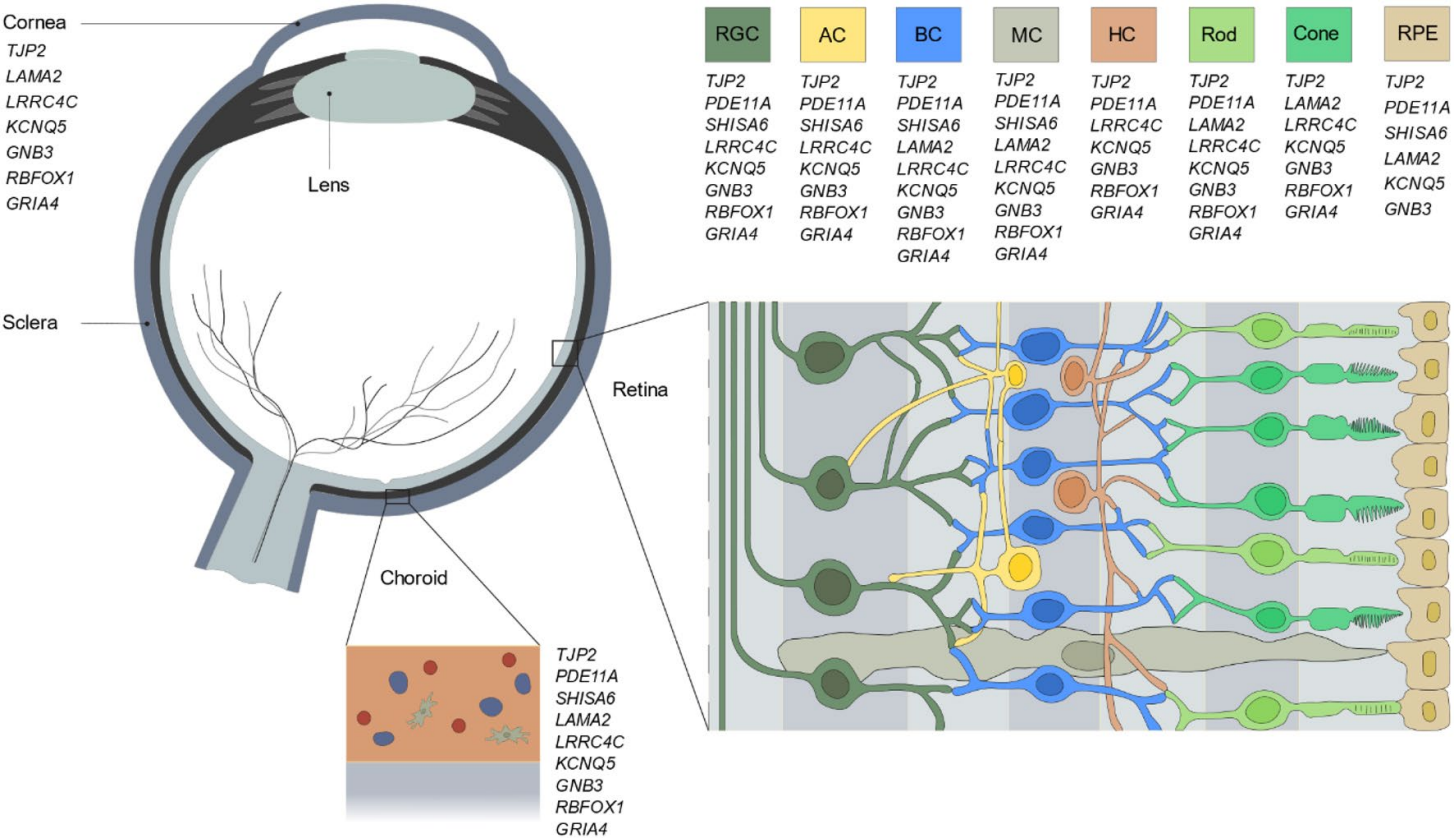
Ocular expression of the prioritized candidate genes. Schematic overview of the expression of the nine candidate genes throughout the human eye. Data on ocular expression was obtained from five independent single-cell RNA sequencing studies^10–12,14,15^. Additional sources were the Human Protein Atlas^13^. Further details on the cell-specific expression of the candidate genes in the cell types of the choroid can be found in Supplementary Data S1. RGC: retinal ganglion cell, AC: amacrine cell, BC: bipolar cell, MC: müller cell, HC: horizontal cell, RPE: retinal pigmented epithelium cell.

We prioritized candidate genes within associated loci from recent GWAS meta-analyses^4,5^. The prioritization was based on both statistical and biological evidence, i.e., (1) effect size (large) and *P*-value (low) of the associated SNPs; (2) expression in ocular tissues; (3) involvement in pathways described in the literature; (4) the existence of a zebrafish ortholog; and additionally, (5) regional visualization of the locus by assessing the locuszoom plots (i.e., the genomic position of the association signal relative to the candidate genes and the position of genes in the region^9^). The final selection consisted of nine top candidate genes; *TJP2*, *PDE11A*, *SHISA6*, *LAMA2*, *LRRC4C*, *KCNQ5*, *GNB3*, *RBFOX1*, and *GRIA4* (see Supplementary Data S1). The cell-specific expression of the nine candidate genes was retrieved from published datasets^10–13^, see Methods section. A summary of the ocular distribution of the genes can be found in Fig. 1 and Supplementary Data S1. According to the retrieved data, all nine genes are expressed throughout the neural retina^10–13^ while expression of *TJP2*, *PDE11A*, *SHISA6*, *LAMA2*, *KCNQ5*, and *GNB3* is also found in RPE cells^11,14^. Moreover, all candidate genes are expressed in cells throughout the choroid in: endothelial cells (*TJP2*, *PDE11A*, *LAMA2*, *LRRC4C*, *GNB3*); pericytes (*TJP2*, *LAMA2*, *GNB3*, *GRIA4*); melanocytes (*TJP2*, *KCNQ5*, *RBFOX1*); fibroblasts (*TJP2*, *PDE11A*, *SHISA6*, *LAMA2*, *LRRC4C*, *GNB3*, *RBFOX1*, *GRIA4*); and immunocompetent cells (*TJP2*, *PDE11A*, *LAMA2*, *RBFOX1*)^11,14^. *TJP2*, *LAMA2*, *LRRC4C*, *KCNQ5*, *GNB3*, *RBFOX1*, and *GRIA4* are additionally expressed in the cornea^15^.

### Expression of candidate genes in the zebrafish eye and generation of mutant zebrafish

**Figure 2:**
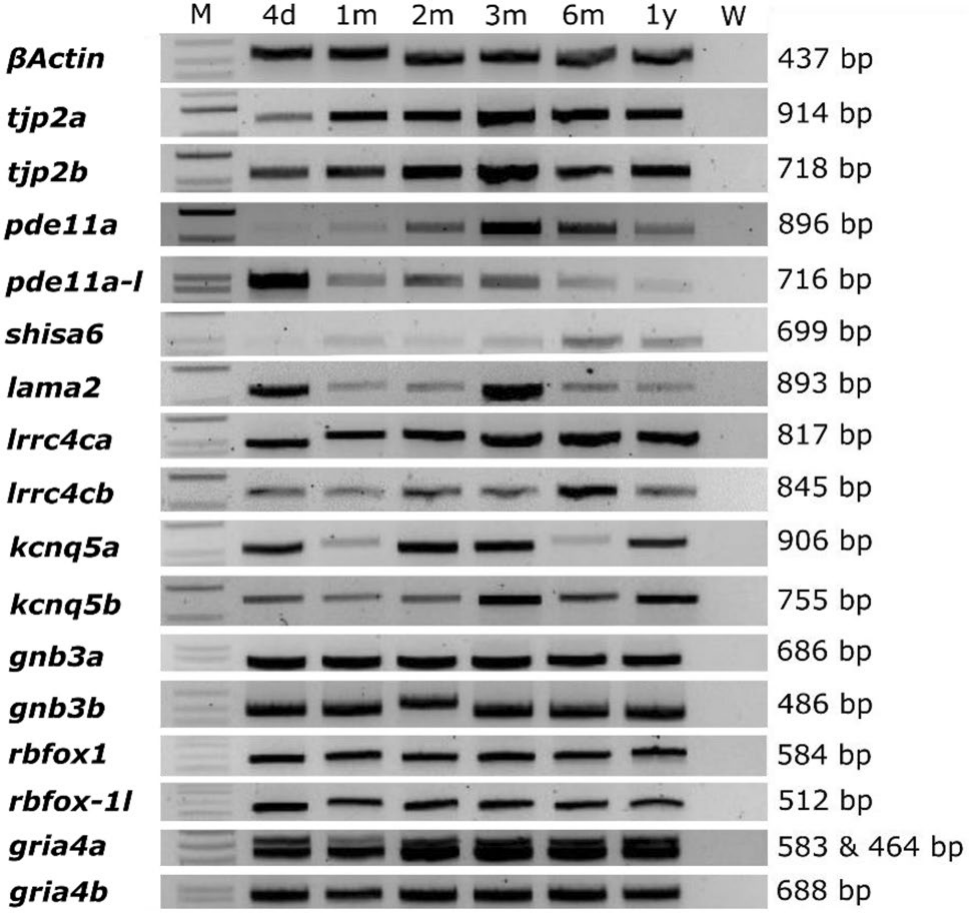
Orthologs of candidate genes are expressed in the zebrafish eye. Agarose gel loaded with RT-PCR product of isolated RNA extracted from zebrafish eyes between 4 days and 1 year post-fertilization. Orthologues genes of human myopia candidate genes showed a continuous expression throughout time. B-actin was used as a positive control. See Supplementary Data S2 for additional information on primers and anticipated product sizes. The figure depicts a grouping of separate gels run for each gene. Full agarose gels are shown in Supplementary Information File 2. M: marker (1 KB + ladder), W: water control, d: days, m: month(s), y: year.

**Figure 3:**
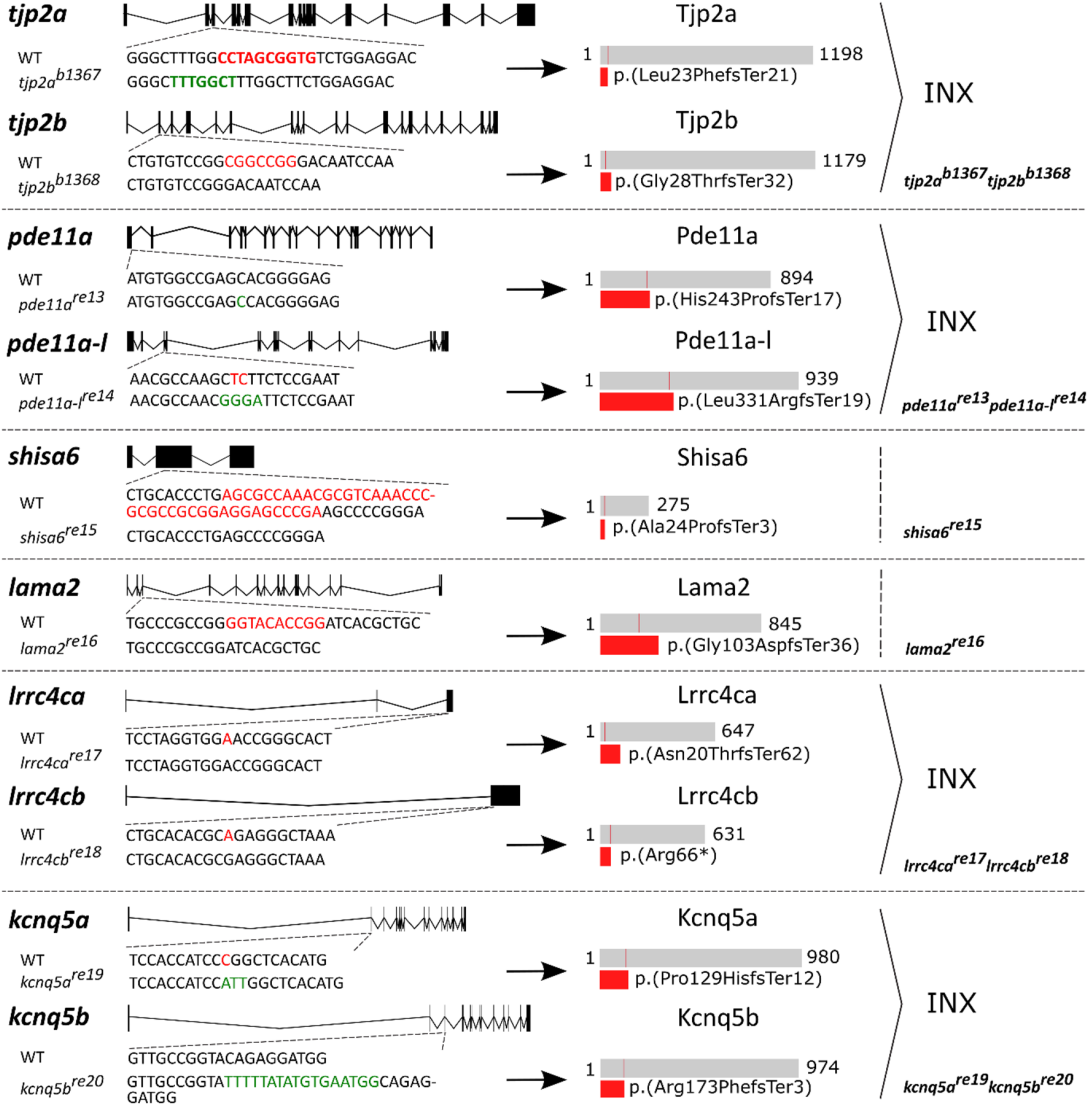
Overview of induced mutations and their predicted effect on protein. Structural representation of the deletions and/or insertions induced by CRISPR-cas9 in the six successfully generated mutant lines. Per gene panel, the dotted lines points to the position of the mutation relative to the gene. Deletions (red) are shown in the WT sequence and insertions (green) in the mutant sequence. A schematic representation of the predicted WT protein (grey) and truncated mutant protein (red) are shown on the right side of the arrow. The region that is predicted to be conserved between the WT and mutant protein is depicted in the WT protein (thin red line). For genes with two orthologues the double mutant generated from crossing the single mutants is depicted on the lower right. WT: wild-type, INX: incross. See Supplementary Data S3 for more detailed information.

Due to a partial genome duplication in teleost fish^16^, 7 out of 9 candidate genes were represented by two zebrafish orthologs; only *SHISA6* and *LAMA2* had one ortholog gene in zebrafish. To confirm gene expression in zebrafish ocular tissue, an Reverse Tanscriptase PCR (RT-PCR) was performed on whole zebrafish eyes at the following developmental stages: 4 days post-fertilization (dpf); 1, 2, 3, 6 months post fertilization (mpf); and 1 year. All studied genes were found to be expressed in the zebrafish eye throughout stages (Fig. 2, Supplementary Data S2). The CRISPR-cas9 system was used to generate mutant zebrafish lines for each fish ortholog of the nine (human) candidate genes (Supplementary Data S3). Generated lines were named according to the Zebrafish Information Network (ZFIN) nomenclature. For each line the induced frameshift mutations led to a premature stop codon, predicted to lead to a truncated protein. Single mutants were created for the genes with one zebrafish ortholog (i.e., *shisa6* and *lama2*): *shisa6*^*re15*^ and *lama2*^*re16*^. Generation of *gria4a* and *gria4b* lines failed due to inability to introduce mutations and perform genotyping of the target region. Double mutants were generated for the remaining six genes with two orthologs in zebrafish: *tjp2a*^*b1367*^*tjp2b*^*b1368*^*, pde11a*^*re13*^*pde11a-like*^*re14*^*, lrrc4ca*^*re17*^*lrrc4cb*^*re18*^*, kcnq5a*^*re19*^*kcnq5b*^*re20*^*, gnb3a*^*re21*^*gnb3b*^*re22*^*, and rbfox1*^*re23*^*rbfox1-like*^*re24*^ (see Supplementary Data S3 for details on the induced mutations). The *gnb3a*^*re21*^*gnb3b*^*re22*^ and *rbfox1*^*re23*^*rbfox1-like*^*re24*^ mutants appeared non-viable after 14 dpf. Therefore, six candidate genes (*shisa6, lama2, tjp2, pde11a, lrrc4c, and kcnq15*) were further characterized. Figure 3 shows a graphic overview of the successfully generated mutants, the mutations, and the predicted truncated proteins.

### SD-OCT assessment showed an increased axial length in three mutant lines

We measured axial length in all six mutant lines using Spectral Domain Optical Coherence Tomography (SD-OCT). To isolate intraocular changes in biometry from potential extraocular changes induced by the generated mutations, we studied mutant fish (n = 20 eyes) with equal body lengths (max variation < 1%) as WT fish (Supplementary Fig. S1).

**Figure 4:**
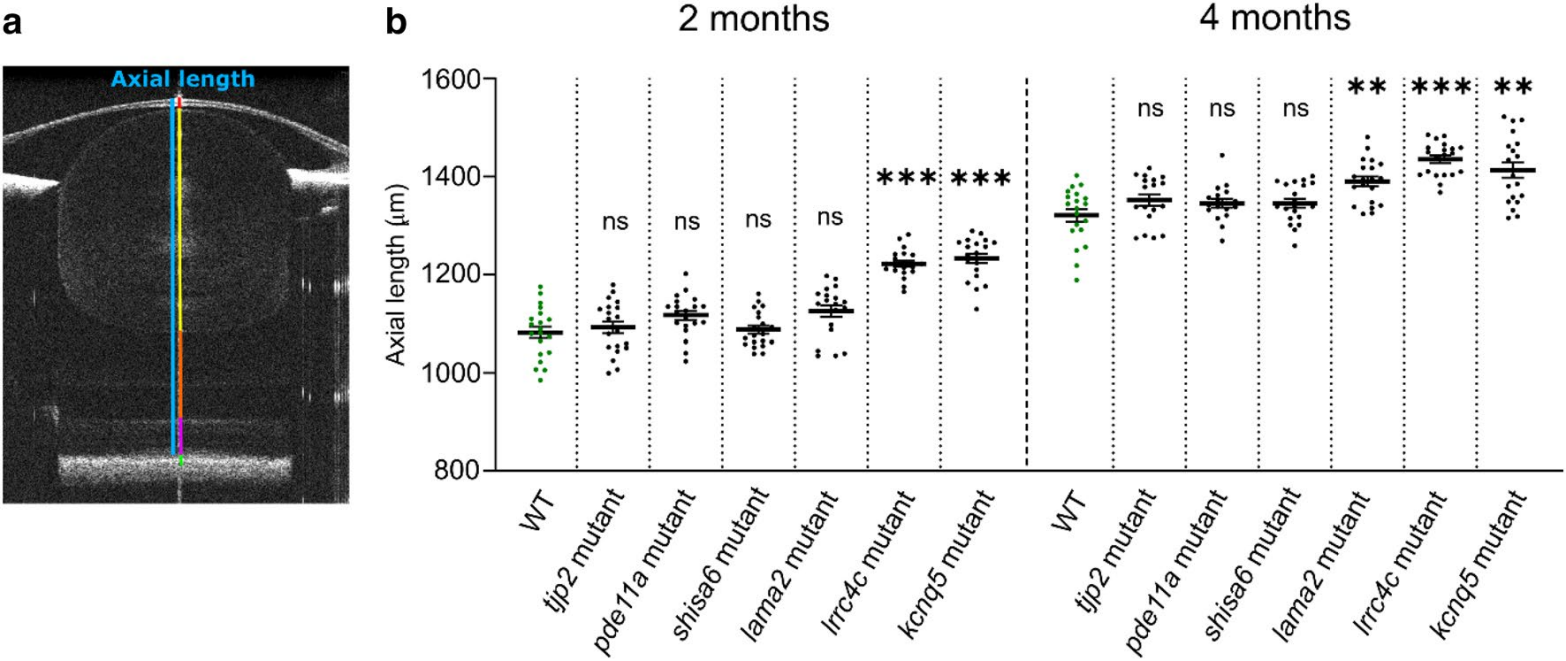
The *lama2*^*re16*^, *lrrc4ca*^*re17*^*lrrc4cb*^*re18*^, and *kcnq5a*^*re19*^*kcnq5b*^*re20*^ mutants showed an increased axial length. (**a**) Single B-scan image of a typical 4mpf WT zebrafish eye. The axial length (blue) spans from the apical part of the corneal epithelium to the anterior border of the RPE. The gradient refractive index of the spherical zebrafish lens was used as a correction factor to acquire this image (see “Methods”). Individual compartments: cornea and anterior chamber (red), lens (yellow), vitreous chamber (orange), neural retina (magenta) and RPE (green). (**b**) Axial length of size-matched 2 and 4mpf zebrafish measured by spectral-domain optical coherence tomography. At 2mpf an increase in axial length was found for *lrrc4c* mutant (Effect size = 140.0 μm, *p* < 0.0001) and *kcnq5* mutant (Effect size = 151.0 μm, *p* < 0,0001) relative to WT fish. At 4mpf a relative increase was found for *lama2* mutant (Effect size = 69.00 μm, *p* = 0.0083), *lrrc4c* mutant (Effect size = 114.0 μm, *p* < 0.0001), and *kcnq5* mutant (Effect size = 92.00 μm, p = 0.0044). See Supplementary Data S4 for all data. Error bars: SEM. Significance: ns = not significant, *p < 0.05, **p < 0.01, ***p < 0.001. Mpf: months post-fertilization, RPE: retinal pigmented epithelium, WT: wild-type, *tjp2* mutant: *tjp2a*^*b1367*^*tjp2b*^*b1368*^, *pde11a* mutant: *pde11a*^*re13*^*pde11a-like*^*re14*^, *shisa6* mutant: *shisa6*^*re15*^, *lama2* mutant: *lama2*^*re16*^*, lrrc4c* mutant: *lrrc4ca*^*re17*^*lrrc4cb*^*re18*^, *kcnq5* mutant: *kcnq5a*^*re19*^*kcnq5b*^*re20*^, SEM: Standard error of the mean.

At 2 mpf, a significant increase in axial length was found for two mutant lines: *lrrc4ca*^*re17*^*lrrc4cb*^*re18*^ (Effect size = 140.0 µm, *p* < 0.0001) and *kcnq5a*^*re19*^*kcnq5b*^*re20*^ (Effect size = 151.0 µm, *p* < 0,0001), relative to WT controls (Fig. 4, Supplementary Data S4). At 4 mpf, a relative increase was also found for the *lama2*^*re16*^ (Effect size = 69.00 µm, *p* = 0.0083) mutant; and the larger axial length persisted for *lrrc4ca*^*re17*^*lrrc4cb*^*re18*^ (Effect size = 114.0 µm, *p* < 0.0001) and *kcnq5a*^*re19*^*kcnq5b*^*re20*^ (Effect size = 92.00 µm, *p* = 0.0044) mutants (Fig. 4, Supplementary Data S4). No significant changes in axial length were found for the *shisa6*^*re15*^, *tjp2a*^*b1367*^*tjp2b*^*b1368*^, and *pde11a*^*re13*^*pde11a-like*^*re14*^ mutants at either time period.

### Refractive status of the mutant zebrafish lines

**Figure 5:**
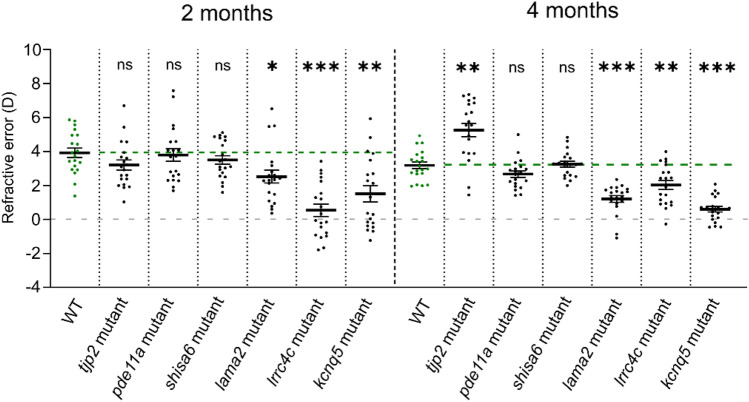
Mutants with increased axial length show a myopic shift in refractive status. Refractive status measured by eccentric photorefraction. The *lama2*, *lrrc4c*, *kcnq5* mutants showed a myopic shift in refractive status at 2mpf (*lama2* mutant: effect size = − 1.4D, *p* = 0.018; *lrrc4c* mutant: effect size = − 3.4D, *p* < 0.0001; *kcnq5* mutant: effect size = − 2.4D, *p* = 0.003) and 4mpf (*lama2* mutant: effect size = − 2.0D, *p* < 0.0001; *lrrc4c* mutant: effect size = − 1.2D, *p* = 0.003; *kcnq5* mutant : effect size = − 2.6D, *p* < 0.0001), relative to WT (n = 20 eyes per timepoint). The tjp2 mutant showed a hyperopic shift at 4mpf (effect size =  + 2.1D, *p* = 0.001). See Supplementary Data S5 for all data. Error bars: SEM. Significance: ns = not significant, *p < 0.05, **p < 0.01, ***p < 0.001. Mpf: months post-fertilization, WT: wild-type, *tjp2* mutant: *tjp2a*^*b1367*^*tjp2b*^*b1368*^, *pde11a* mutant: *pde11a*^*re13*^*pde11a-like*^*re14*^, *shisa6* mutant: *shisa6*^*re15*^, *lama2* mutant: *lama2*^*re16*^, *lrrc4c* mutant: *lrrc4ca*^*re17*^*lrrc4cb*^*re18*^, *kcnq5* mutant: *kcnq5a*^*re19*^*kcnq5b*^*re20*^, SEM: Standard error of the mean.

To explore whether the changes in axial length resulted in alterations in refractive status, a custom eccentric photorefraction set-up was used as described previously^7,8^. The WT control fish (n = 20 eyes) showed a positive baseline refractive status (Fig. 5, Supplementary Data S5). This has also been found in previous studies, and has mainly been attributed to the small eye retinoscopic artifact^7,8,17–19^. In agreement with our biometric data, a myopic shift in refractive status was found at 2 mpf for *lrrc4ca*^*re17*^*lrrc4cb*^*re18*^ (effect size = − 3.4D, *p* < 0.0001) and for *kcnq5a*^*re19*^*kcnq5b*^*re20*^ (effect size = − 2.4D, *p* = 0.003) relative to WT controls (Fig. 5, Supplementary Data S5). The *lama2*^*re16*^ mutant showed a significant myopic refraction at 2 mpf as well (effect size = − 1.4D, *p* = 0.018). Likewise, a myopic shift was also detected at 4 mpf for *lrrc4ca*^*re17*^*lrrc4cb*^*re18-/-*^ (effect size = − 1.2D, *p* = 0.003), *kcnq5a*^*re19*^*kcnq5b*^*re20*^ (effect size = − 2.6D, *p* < 0.0001) and *lama2*^*re16*^ (effect size = − 2.0D, *p* < 0.0001) (Fig. 5, Supplementary Data S5).

No changes in refractive status were found at 2 nor at 4 mpf for the *pde11a*^*re13*^*pde11a-like*^*re14*^ and *shisa6*^*re15*^ mutants. However, the *tjp2a*^*b1367*^*tjp2b*^*b1368*^ mutant showed a hyperopic shift in refractive status (effect size =  + 2.1D, *p* = 0.001) at 4 mpf (Fig. 5, Supplementary Data S5). In contrast to the three myopic mutants, the *tjp2a*^*b1367*^*tjp2b*^*b1368*^ mutant did not show significant changes in axial length. In the OCT data, however, we observed an increase in lens diameter of the *tjp2a*^*b1367*^*tjp2b*^*b1368*^ at 4 mpf relative to WT lenses (Δ increase = 26.70 µm, *p* = 0.035) (Supplementary Data S4). We hypothesized that the measured hyperopic refractive change of the mutants was due to changes in the lenticular shape with, as a consequence, a decreased lens curvature. To assess this hypothesis, we compared 4 mpf isolated ex-vivo lenses (per group n = 10 fish, n = 20 lenses), and found a circularity = 1 for both mutant and WT lenses, which suggests a homogeneous increase in lens diameter and volume in the *tjp2a*^*b1367*^*tjp2b*^*b1368*^ mutant (see Supplementary Fig. S2a, b and Methods). Next, we used the 4 mpf SD-OCT data and measured the changes in curvature of the anterior lenticular hemisphere. Here we found a significant decrease in curvature in the lenses of the *tjp2a*^*b1367*^*tjp2b*^*b1368*^ mutants (Supplementary Fig. S2c). This decreased curvature may contribute to the increased focal length and the detected hyperopic shift in refractive error.

### Topographic overview of selected genes leading to a refractive error phenotype

**Figure 6:**
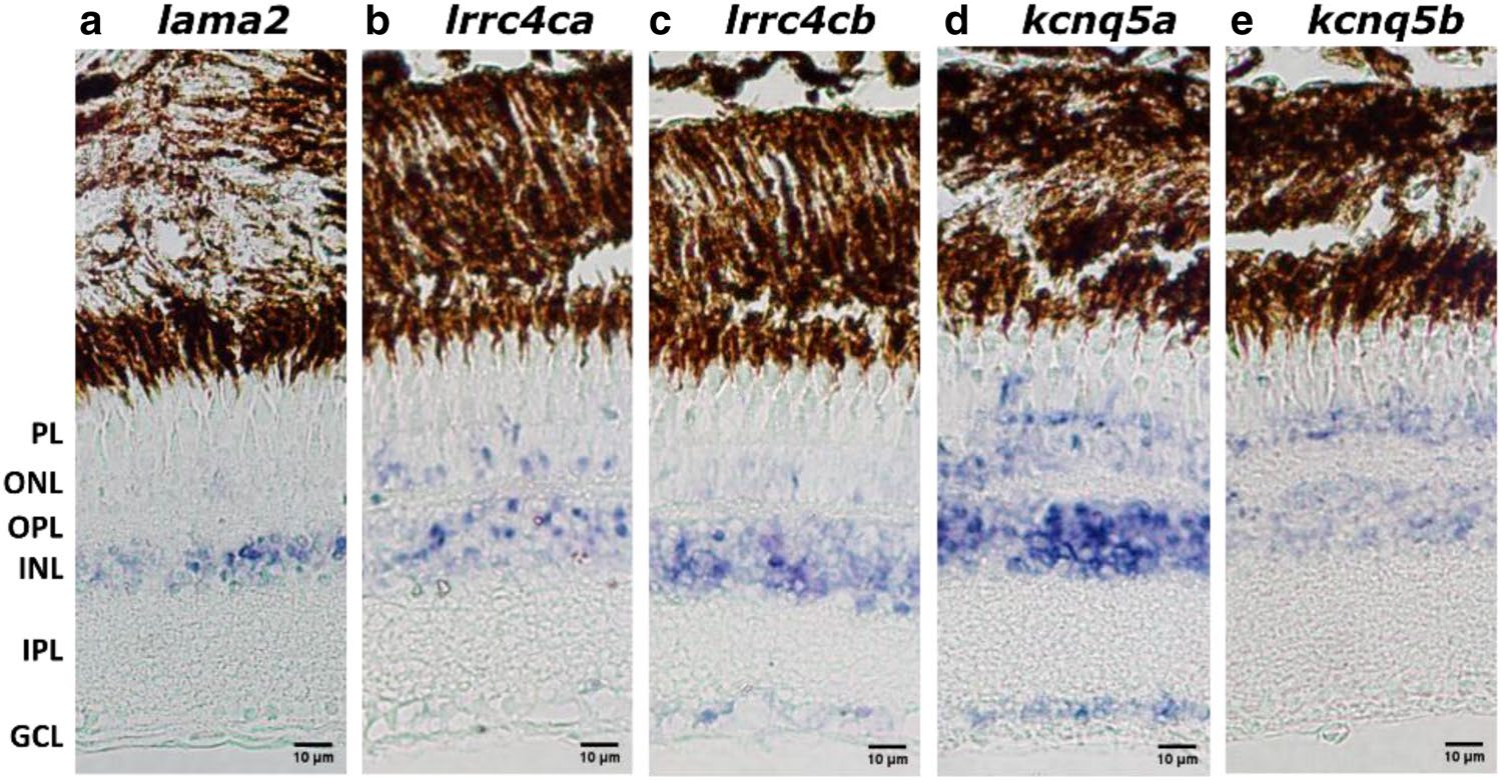
Ocular distribution of *lama*, *lrrc4ca*/*lrrc4cb* and *kcnq5a*/*kcnq5b* in zebrafish. In situ hybridization showing the distribution of *lama2, lrrc4ca*, *lrrc4cb*, *kcnq5a* and *kcnq5b* throughout the 1-year-old WT zebrafish retina. NBT/BCIP, in purple, was used to visualize the probes. Lama2 expression was mainly found in the INL and also modestly in the ONL (**a**). *Lrrc4ca* and *lrrc4cb* expression was found in both ONL and INL (**b**–**c**) whilst *lrrc4ca* expression was also detected in the PL (**b**) and *lrrc4cb* expression in the GCL (**c**). *Kcnq5a* expression was found in the PL, ONL, INL and GCL (**d**) whilst *kcnq5b* expression was found in only the ONL and the INL (**e**). Scale bars: 10 μm. WT: Wild Type, PL: photoreceptor layer, ONL: outer nuclear layer, OPL: outer plexiform layer, INL: inner nuclear layer, IPL: inner plexiform layer, GCL: ganglion cell layer.

To characterize the retinal role of *lama2*, *lrrc4ca*, *lrrc4cb*, *kcnq5a*, and *kcnq5b,* we studied the expression and topographic distribution using in-situ hybridization in one year old WT fish. Expression of *lama2* was predominantly found in the inner nuclear layer (INL) and more modest in the outer nuclear layer (ONL) (Fig. 6a). Expression of both *lrrc4ca* and *lrrc4cb* was observed in the ONL and INL (Fig. 6b,c). *lrrc4ca* was also modestly detected in the photoreceptor layer (PL) whereas *lrrc4cb* was additionally found in the ganglion cell layer (GCL). k*cnq5a* was expressed in the GCL, INL, ONL, and PL (Fig. 6d) and *kcnq5b* was limited to the INL and ONL (Fig. 6e). The ISH showed that these myopia candidate genes are all expressed throughout zebrafish retina.

## Discussion

Even though GWASs have successfully provided insights into the genetic architecture of complex traits such as refractive error, GWASs do not provide information regarding the causal variant or its functional role. In this study, we present a candidate-gene approach study in which we aim to validate and explore the functional role of RE-candidate genes in the eye using mutant zebrafish models. We prioritized nine candidate genes identified in previous GWASs of RE and used CRISPR/cas9 technology to generate mutant zebrafish. Ocular screening for refractive error was possible in six mutant lines, and in three of those, we observed a myopic phenotype: *lama2*^*re16*^, *lrrc4ca*^*re17*^*lrrc4cb*^*re18*^*,* and *kcnq5a*^*re19*^*kcnq5b*^*re20*^. In our ISH studies we showed that *lama2*, *lrrc4ca, lrrc4cb, kcnq5a and kcnq5b* are expressed throughout the zebrafish retina in accordance with our search in published scRNA-seq databases, and supporting previous notions that suggest that the retina (and its intricate signaling cascades) contributes as a whole in the development of both emmetropia and ametropia. Our study provides new in vivo models that will help dissecting the pathways leading to RE development.

The variant rs12193446, located in intron 58 of the human *LAMA2* gene (NM_000426.4), has showed statistically the strongest association with RE, with a *P*-value of = 9.87 × 10^−328^ in the largest GWAS meta-analysis^4,5^. It is unclear if this deep intronic variant is truly causal since its potential functional effect has not been elucidated yet. However, in our candidate-gene approach we found an increase in axial length at 4 mpf in the *lama2*^*re16*^ mutant, whereas a myopic shift in refractive error was detected at both 2 and 4 mpf. The difference in onset of detectable changes by SD-OCT and photorefraction can be due to the sensitivity and methodological differences between these two methods. In our in-situ hybridization study, we observed that zebrafish *lama2* was predominately expressed in the INL. Previous studies on avian and rodent tissues also reported expression in the retinal ganglion cells (RGC)^20^ and vasculature^21,22^.

In previous zebrafish studies, mutations in *lama2* led to severe muscular dystrophy phenotypes and growth abnormalities between 8 and 15 dpf^23–25^. In contrast to these previously generated mutant lines, our *lama2*^*re16*^ mutants showed no swimming abnormalities, macroscopically visible growth deficits or early death, however, a more comprehensive study into the muscle fiber composition would be needed to exclude degeneration of muscle tissue. The *lama2*^*re16*^ mutant used in this study contains a frameshift mutation and predicted stop gain in an early exon (#3), expected to lead to nonsense-mediated decay or at least early termination and a severely truncated Lama2 protein. Whereas the previously reported *lama2* models carry mutations located in highly conserved amino acid residues that map to the globular domain of lama2 and that are known to cause muscular dystrophy in humans. Note that the absence of a severe early onset muscular dystrophy phenotype in the *lama2*^*re16*^, created the possibility to focus on the intraocular consequences of the mutation, i.e., RE development. In humans, mutations in *LAMA2* as well as mutations in the two related genes: *DAG1* and *ITGA7*, both encoding LAMA2 receptors, are known to cause muscular dystrophy^22,26–29^. All three genes were significantly associated with RE in previous GWAS meta-analyses^5^. The relationship between the common variants in *LAMA2*, *DAG1* and *ITGA7* that are associated with myopia and the mutations in these same genes causing muscular dystrophy is not clear. Studies have shown a role of laminins, including laminin alfa-2 and the integrity of the retinal vascular basement membrane. Furthermore, it is known that patients with bi-allelic pathogenic variants in *LAMA1* (causing Poretti–Boltshauser syndrome, OMIM #615960) develop high myopia as one of the symptoms. The myopic phenotype after depletion of *lama2* in zebrafish, together with the strong evidence from GWAS studies support the notion that human *LAMA2* contributes to myopia.

Rs11602008, an intronic variant in the *LRRC4C* gene (NM_001258419.2), has consistently been associated with RE in multiple GWAS meta-analyses^4,5,30^. This variant has been annotated to the *LRRC4C* gene encoding the netrin-G1 ligand (NGL-1). NGL-1 is a transmembrane protein and together with its presynaptic ligand Netrin-G1 are known to promote neurite outgrowth of developing thalamic neurons^31,32^. Furthermore, *Lrrc4c*/NGL-1 deletion in male mice suppresses hippocampal excitatory synapse development and function^33^. Although the retinal function of *LRRC4C*/NGL-1 has not been described yet, we observed in the retrieved scRNA-seq databases and our ISH studies that human *LRRC4C* and zebrafish *lrrc4c* genes are both expressed throughout the neural retina. Hence, the potential role of *LRRC4C* in the retina may, similar to the thalamus, also be sought in the direction of axon guidance during development, serving a role in controlling retinal wiring. In zebrafish, we showed that a potential loss of *lrrc4c* leads to axial elongation and a myopic shift in refractive status. Defects in retinal signaling induced by the loss of zebrafish *lrrc4c*/Ngl-1 and potentially the retinal wiring in the developing mutant zebrafish may disturb the emmetropization process. The precise mechanism in which an alteration in the retina-to-sclera signaling cascade, due to potential lack of *lrrc4c,* may lead to axial elongation remains to be elucidated. Studies exploring the transcriptomic changes downstream *lrrc4c* will provide more insights into this process.

The rs7744813 SNP in intron 1 of *KCNQ5* (NM_019842.4) has been associated with myopia by multiple independent GWAS meta-analyses^4,5,30^. Moreover, rs7744813 and four other *KCNQ5* polymorphisms have also been associated with high myopia^34^. In this study, we described that human *KCNQ5* and zebrafish *kcnq5a/kcnq5b* are widely expressed throughout the retina and that the potential loss of *kcnq5* leads to myopia in zebrafish. *KCNQ5* encodes a voltage-gated potassium channel named Kv7.5. It has been shown that *KCNQ5*/kv7.5 is mediating M-type current in the brain^35,36^, retinal pigmented epithelium (RPE) and neural retina^37,38^. Yang et al. 2021 showed a potential relation between *KCNQ5* and myopia development in form-deprived guinea pigs. The authors reported that retinal *KCNQ5* was significantly downregulated, and proposed that this was the result of increasing potassium concentrations or decreasing M-type potassium current densities in the RPE cells of form-deprived guinea pig eyes^39^. In our study, however, the ocular axial elongation and corresponding (myopic) shift in refractive error is observed as result of the potential lack of *kcnq5* in zebrafish. The order of events in the guinea pig model may therefore have been: form-deprivation myopia; followed by downregulation and impairment of the M-type potassium current; leading to axial ocular growth. Another study proposed that changes in expression patterns or the function of *KCNQ5* affects the timing of retinal bipolar cell signals generated in response to photoreceptor light-evoked responses^40^. These altered bipolar cell responses may enhance the susceptibility to myopia. Our findings in zebrafish support the findings reported in other animal models, which show evidence for a relationship between *kcnq5* and myopia. Future studies using *KCNQ5* (Kv7.5)-selective potassium channel openers or inhibitors may lead to the discovery of new myopia treatments.

Besides the myopic phenotype observed in the *lama2*^*re16*^, *lrrc4ca*^*re17*^*lrrc4cb*^*re18*^*,* and *kcnq5a*^*re19*^*kcnq5b*^*re20*^ mutants, we found that the *tjp2a*^*b1367*^*tjp2b*^*b1368*^ mutants showed a hyperopic shift in refractive status at 4 mpf, however, without a corresponding decrease in axial elongation. This may be a direct effect of the potential loss of tight junctions in lens tissue as *tjp2a* and *tjp2b* seem to be moderately expressed in the lenses of larval zebrafish^41,42^. Zebrafish cannot accommodate due to a vestibular retractor lentis^43–45^, also, the zebrafish cornea does not contribute to the optical power in water^46–48^. The refractive error is, therefore, most likely induced by an increased focal length, i.e., the increased lens diameter and decrease in curvature/sphericity. To assess our hypothesis, we studied the relative differences between WT and *tjp2a*^*b1367*^*tjp2b*^*b1368*^ mutant lenses; which brings limitations, as we did not perform absolute calculations of the optical power. Ideally, the front and back of the lens should both be measured, however in the SD-OCT scans, the back of the lens is deformed due to the lens effect, leading to image deformation. Nevertheless, the front and back of the lens are assumed to be equally shaped due to the perfect sphericity we measured ex-vivo. A final limitation of our lens assessment is that we only considered changes in the outer cortical region and epithelium and not the potential changes in the optical properties of deeper cortical and nuclear layers. In this study, we identified a potential lenticular function of *tjp2a* and *tjp2b*, however, future studies are needed for an in-depth characterization of both the molecular and optical changes in the *tjp2a*^*b1367*^*tjp2b*^*b1368*^ mutant lens.

In our screening, we did not observe an ocular phenotype in the *pde11a*^*re13*^*pde11a-like*^*re14*^ and *shisa6*^*re15*^ mutants. However, a limitation of this study is the lack of antibodies specific to the studied zebrafish proteins, which limited our opportunity to confirm by western blot or immunohistochemistry the effect of the generated mutant alleles at the protein level. Therefore, based on these results, we cannot exclude these genes as potentially involved in regulating eye size.

Interpreting GWAS findings is challenging and requires not only the identification of the causal variant but also the identification of the molecular effect of the variant, the role of the implicated genes, and the relevant cell types and pathways. In this study, we present a candidate-gene approach in response to the lack of functional studies exploring the potential role of candidate genes on refractive error development. We prioritized nine candidate genes, screened six mutant zebrafish models, and found a myopic phenotype in three mutant lines (*lama2*^*re16*^, *lrrc4ca*^*re17*^*lrrc4cb*^*re18−/−*^, and *kcnq5a*^*re19*^*kcnq5b*^*re20*^). The myopic phenotype was characterized by an increase in axial length and negative shift in refractive status. These findings indicate that myopic refractive errors can be induced by depletion of individual strongly associated candidate genes selected from GWAS. These single candidate genes, when depleted, leading to significant changes in refractive status is interesting, considering the consensus that complex myopia is induced by an epistasis of SNPs and genes. Our findings show that these three candidate genes, when depleted, independently contribute to axial length determination. The myopic *lama2*^*re16*^, *lrrc4ca*^*re17*^*lrrc4cb*^*re18-/-*^, and *kcnq5a*^*re19*^*kcnq5b*^*re20*^ mutants can be used to further study the signaling cascade underlying myopic axial elongation and the development of myopia interventions.

## Methods

### Lookup of candidate genes in published transcriptomic datasets

Retinal expression of the candidate genes was assessed using three independently published single-cell RNA sequencing (scRNA-seq) datasets of the adult human retina^10–12^ and the Human Protein Atlas^13^. Outside the neural retina we retrieved scRNA-seq data from the retinal pigmented epithelium (RPE) and choroid^14^, and cornea^15^. Single cell expression studies on human scleral and lens tissue have not yet been published; therefore, these tissues were not considered in this assessment.

### Fish lines and housing

The mutant lines were generated using CRIPSR-cas9 in WT-AB zebrafish. Single guide RNAs (sgRNA) were designed (Supplementary Data S3) and synthesized according to the CRISPRscan protocol (http://www.crisprscan.org)^49^. The oligo for sgRNA synsthesis included respectively a: 5′ T7 promotor sequence (aattaatacgactcactata), target site sequence, and a loop sequence (gttttagagctagaaatagc). This oligo was combined with a complementary loop oligo (AAAAGCACCGACTCGGTGCCACTTTTTCAAGTTGATAACGGACTAGCCTTATTTTAACTTGCTATTTCTAGCTCTAAAAC) that includes the RNA loop for Cas9 recognition. PCR was performed to generate the DNA guide oligo's followed by transcription of the sgRNAs by the T7 megascript kit (ThermoFisher). sgRNAs and Cas9 protein were combined, and 1 nl was injected (final concentration of 1500 pg/nl) into WT AB eggs at the one-cell stage^49–51^. Further details of the protocol can be found in Vejnar et al. 2017. Induced mutations were validated by Sanger sequencing, and founders were crossed to generate homozygous knock-out fish. Double knock-outs were generated for the genes with multiple orthologs in zebrafish (Supplementary Data S3). The *tjp2a*^*b1367*^ and *tjp2b*^*b1368*^ lines were generated previously at the University of Oregon^50^. These lines were crossed to generate the *tjp2a*^*b1367*^*tjp2b*^*b1368*^ line used in this study. Fish lines were raised in tanks with matched population sizes. For spectral domain optical coherence tomography (SD-OCT) and photorefraction zebrafish were anesthetized using a 0.016% tricaine methane sulfonate solution (MS222, Sigma Aldrich), buffered to pH = 7. All animals were treated in accordance with the Dutch animal welfare legislation and the guidelines from the experimental animal health care center (EDC: Experimenteel Dier Centrum) of the Erasmus Medical Center Rotterdam, The Netherlands, and in accordance with the European Commission Council Directive 2010/63/EU (CCD approval, license AVD 1010020186907). In this study we have followed the reporting recommendations described in the ARRIVE guidelines. All zebrafish were exposed to a 14-h light: 10-h dark cycle at 28.5 °C.

### RNA isolation from zebrafish eyes and expression analysis

Enucleated eyes were collected from 4 dpf (n = 20 heads), 1 mpf (n = 8 eyes), 2 mpf (n = 6 eyes), 3 mpf (n = 5 eyes), and 12 mpf (n = 4 eyes) WT zebrafish. Lenses were removed under a stereomicroscope (Leica m80), and tissue was collected and frozen in liquid nitrogen. 500 µl of Trizol reagent (Ambion) was used to extract RNA, followed by homogenization using a handheld homogenizer (Pro 200, Pro scientific Inc.) for 2 × 5 s. The homogenate was left at room temperature (RT) for 5 min, and 200 µl of chloroform (Sigma Aldrich, ≥ 99%) was added. The samples were incubated at RT for 15 min and centrifuged at maximum RPM for 15 min at 4 °C. Next, the upper fraction was collected, and RNA was extracted using the RNeasy Mini Kit (Qiagen). The extracted RNA was quantified using a Nanodrop (DS-11 Series Spectrophotometer/fluorometer, DeNovix) and used to synthesize cDNA by the iScript cDNA Synthesis kit (BioRad). The cDNA transcripts were confirmed by PCR and agarose gel electrophoresis, using the primer sets shown in Supplementary Data S2.

### Spectral domain optical coherence tomography (SD-OCT)

SD-OCT measurements and analysis were performed as previously described by Quint et al.^8^. 10 fish (20 eyes) of each genotype were measured at 2 and 4 mpf. Mutant fish included in the study all had < 1% body length variation relative to WT fish (Supplementary Fig. S1), ruling out variability in ocular metrics due to significant changes in body size. A 900 nm SD-OCT Ganymede system (Thorlabs) was used. The field of view was set to 1.7 × 1.7 × 2.2 mm with a pixel depth of 2 µm in the Z-direction. Custom MATLAB software was used for the analysis of the ocular metrics, as described previously^8^, and the dimensions of the ocular components were corrected using the specific refractive index of each eye component; the cornea 1.33^46–48^, the lens (gradient refractive index) 1.4^46–48,52–54^, the anterior and vitreous chamber 1.34, and the retina 1.38^48,55,56^.

### Eccentric photorefraction

The refractive status of the zebrafish eyes was determined by eccentric infrared photorefraction. Further details of this protocol can be found elsewhere^8^. The analysis was done by custom C +  + software. Mutant and control lines were measured at 2 and 4 mpf. The slope of the brightness gradient was determined by 100 independent measurements and averaged. This value was converted into refractive error in Diopter by calibration with ophthalmic lenses^8^.

### Lens sphericality and curvature measurements

Lenticular biometry was performed in ex-vivo isolated lenses from 4 mpf WT and *tjp2a*^*b1367*^*tjp2b*^*b1368*^ mutant fish (n = 10 fish, n = 20 lenses). We hypothesized that the measured hyperopic refractive change of the *tjp2a*^*b1367*^*tjp2b*^*b1368*^ mutant was due to changes in the lenticular shape with, as a consequence, a decreased lens curvature. This hypothesis is based on the fact that in zebrafish: (1) the rigid lens cannot accommodate due to a vestigial retractor lentis^43–45^, (2) the full optical power comes from the spherical lens as the contribution of the cornea is negligible in water (refractive index: 1.33)^46–48^, and (3) we observed an increased lens diameter in the *tjp2a*^*b1367*^*tjp2b*^*b1368*^ mutant. However, changes in sphericity cannot be directly determined from the SD-OCT data due to the blockage of infrared light by the iris pigment. To overcome this limitation, we assessed the lens sphericity indirectly by comparing 4 mpf isolated ex-vivo lenses. Lenses were positioned under a light microscope (Olympus DP72) and photographed (Olympus U-TV0.63XC) from 3 different angles. We used the circularity of the great circle (mid-plane), measured from three different angles, as a proxy for the sphericity of each lens (circularity was measured in Fiji (ImageJ)). We observed that the circularity from all three positions was equal to 1 for both the mutant and WT lenses (Supplementary Fig. S2b). This conservation of the circularity/sphericity means that the increase in lens diameter in the *tjp2a*^*b1367*^*tjp2b*^*b1368*^ mutants was equally distributed in all axial directions. This type of homogeneous increase in lens diameter and volume, i.e., when the sphericity stays equal to the WT lenses, leads to a decrease in lens curvature. To confirm this finding, we also directly measured the changes in curvature of the anterior lenticular hemisphere in the 4 mpf SD-OCT data of 4 mpf WT and *tjp2a*^*b1367*^*tjp2b*^*b1368*^ mutants (n = 10 fish, n = 20 lenses). Analysis was done in Fiji (ImageJ) using the Kappa plugin for curvature measurements.

### In situ* hybridization*

Dissected eyes of one-year-old WT zebrafish were stored in 4% Paraformaldehyde (PFA) for two days, fixated using an EFTP tissue processor (Intelsint), and embedded in paraffin prior to sectioning. Embedded eyes were sectioned following standard protocols using a microtome (Microm HM 335 E). Paraffin sections (6 µm) were transferred to Superfrost slides (VWR) and dried at 37 °C. Sections were deparaffinized in xylene for 5 min and 10 min, 100% ethanol for 5 min, 70% ethanol for 10 min, and PBST (1 × Phosphate buffered saline with 0.1% Tween-20) for 5 min. Sections were fixed in 4% PFA for 20 min and washed in PBST for 5 min before pre-hybridization in preheated HYB + buffer (50% Formamide, 5 × SSC (Saline-sodium citrate), 50 µg/ml Heparin, 500 µg/ml tRNA, 0.1% Tween-20, and aqua dest) at 68 °C for 1 h. Sense and anti-sense probes (see Supplementary Data S2) were diluted in HYB + , heated at 95 °C for 2 min, and chilled on ice until needed. Sections were hybridized with the probes overnight at 68 °C in a humidified chamber. Sections were washed in preheated wash-buffer (50/50 HYB- (50% Formamide, 5 × SSC, 0.1% Tween-20, and aqua dest)/0.2 × SSC) at 68 °C for 15 min and in preheated 0.2 × SSC at 68 °C for two times 30 min. Next, sections were washed in respectively 75%; 50%; 25%; 0% 0.2 × SSC in PBST for 15 min, at RT. Sections were blocked for one hour at RT with blocking solution (2% sheep serum, 2 mg/ml Bovine Serum Albumin (Sigma Aldrich) in PBST) followed by incubation with antiDIG-AP antibody (Anti-Digoxigenin-AP, Fab fragments, 1:2000, Roche) diluted in blocking solution (Overnight, 4 °C). Sections were washed in PBST for 3 times 15 min and in Staining buffer NTMT (1 M Tris (pH 9.5), 1 M MgCl2, 5 M NaCl, 10% Tween-20) for 2 times 5 min. Staining solution (12 µl NBT/BCIP (Roche) per 1 ml Staining buffer) was applied to the sections and were incubated in the dark at RT. Incubation time was variable dependent on the probe set, ranging from 1 hour up to 2 days. Sections were respectively washed in PBST for 3 times, 15 min, rinsed in distilled water, hydrated in respectively 95% and 100% ethanol for 1 min each, and finally exposed to xylene for 2 times, 2 min. Slides were mounted with Entellan (Sigma Aldrich) and dried overnight at 37 °C. Imaging was performed on an Olympus DX40 microscope with an Olympus DP73 camera. Olympus cellSens software was used for image processing.

### Statistical analysis and sample size estimation

We analyzed the SD-OCT and photorefraction by fitting a mixed model as implemented in GraphPad Prism 8.0. This mixed model uses a compound symmetry covariance matrix, and is fitted using Restricted Maximum Likelihood (REML). With this model both eyes of the same fish are treated as repeated measures. Outliers were detected by Grubbs’ test (*p* < 0.01) and excluded from the analysis. The body length measurements were analyzed with Welch’s ANOVA, also in GraphPad Prism 8.0.

To estimate the sample size for the SD-OCT and photorefraction assessment, we used the GLIMMPSE software^57^ for repeated measures. Estimated effect sizes (i.e., approximately 4% change in axial length and approximately 6% change in photorefraction) were based on a previous study^8^, alpha-error was set to 0.05 and power to 0.95. Based on these parameters, a minimum size of 20 eyes (10 fish) per group was required (power = 0.953).

## Supplementary Information

Supplementary Information 1.

Supplementary Information 2.

Supplementary Information 3.

Supplementary Information 4.

Supplementary Information 5.

Supplementary Information 6.

## Data Availability

All data generated or analyzed during this study are included in this published article and its supplementary information files. Any additional (raw) data is available from the corresponding author on request.
