# Supplementary material for: Post-GWAS screening of candidate genes for refractive error in mutant zebrafish models
Source: Sci Rep. 2023 Feb 3;13:2017. doi: 10.1038/s41598-023-28944-y (PMC9898536; doi:10.1038/s41598-023-28944-y)

Supplementary Information

Post-GWAS screening of candidate genes for refractive error in mutant zebrafish models.

Wim H. Quint[1,2], Kirke C.D. Tadema[1,2*], Nina C.C.J. Kokke[1,2*], Magda A. Meester-Smoor[1,3], Adam C. Miller[4], Rob Willemsen[2], Caroline C.W. Klaver[1,3,5,6]±, Adriana I. Iglesias[1,2]±

1 Department of Ophthalmology, Erasmus Medical Center, Rotterdam, The Netherlands
2 Department of Clinical Genetics, Erasmus Medical Center, Rotterdam, The Netherlands
3 Department of Epidemiology, Erasmus Medical Center, Rotterdam, The Netherlands
4 Institute of Neuroscience, University of Oregon, Eugene, United States.
5 Department of Ophthalmology, Radboud University Medical Center, Nijmegen, The Netherlands
6 Institute of Molecular and Clinical Ophthalmology Basel, Basel, Switzerland

* K.C.D.T and N.C.C.J.K contributed equally to this work.

± C.C.W.K and A.I.I jointly supervised this work.

Content:

Supplementary Information

- **In situ hybridization protocol**

Supplementary Figures

- **Supplementary Figure S1: Body length.**
- **Supplementary Figure S2. Circularity and curvature of WT and tjp2**
- **Supplementary Figure S3. Full-length gels included in Fig. 2**

Supplementary Information

In situ hybridization protocol

Day 1

- 5 minutes in Xylene
- 10 minutes in Xylene
- 5 minutes in 100% Ethanol
- 10 minutes in 70% Ethanol
- 5 minutes in PBST (1x Phosphate buffered saline with 0.1% Tween)
- Postfix in 4%PFA for 20 minutes (on slide)
- 5 minutes wash in PBST
- Pre-hybridise slides/sections in preheated HYB+ buffer at 68ºC for 1h
- Dilute the probes in HYB+ in PCR tubes. Heat at 95°C for 2 min in a PCR block to denature the RNA probe. Chill on ice immediately to prevent reannealing.
- Hybridise with probe overnight (12-16 hours) at 68ºC on the slide. Apply probe and cover slides with glass cover-slides and incubate in a humidified chamber

Day 2

- Wash slides in preheated wash-buffer (50/50 HYB-/0.2x SSC) at 68ºC 1 x 15min
- Wash slides in preheated 0.2x SSC 68ºC at 2 x 30min
- Wash slides in 75/50/25/0% 0.2x SSC in PBST at RT 15 min each
- Block 1h at RT (2% sheep serum 2 mg/ml BSA in PBST)
- Prepare the antibody dilution with the 2% Roche Blocking Agent Solution. antiDIG-AP antibody 1:2000
- Incubate with the antibody over night at 4˚C (on the slide in a humidified chamber)

Day 3

- Wash 3 x15 minutes in PBST
- Wash in Staining buffer 2 x 5 minutes
    - 1M Tris (pH 9.5) 5 ml
    - 1M $MgCl_2$ 2.5 ml
    - 5M NaCl 1 ml
    - 10% Tween20 0.5 ml
    - Sterile water 41 ml
- Apply staining solution (on the slide) and incubate in the dark at room temperature. The duration depends on the strength of your probe. It can be anywhere from one hour up to several days
    - 12 µl NBT/BCIP per ml Staining Buffer

Day 3 or when the reaction is finished

- Wash 3 x15 minutes in PBST
- 15 dips in water
- 2 x 1 minute incubation in 95% Ethanol
- 2 x 1 minute incubation in 100% Ethanol
- 2 x 2 minutes incubation in Hemo-De (Xylene substitute)
- mounting slides with Entellan and dry overnight

Day 4

- Image under microscope

HYB+ buffer recipe:

- 50% Formamide
- 5x Saline Sodium Citrate (SSC)
- 50 µg/ml Heparin
- 500 µg/ml tRNA
- 0.1% Tween-20
- dH_2O
- Store at -20 C

To make HYB-, exclude the Heparin and tRNA

Supplementary Figures

Supplementary Figure S1. Body length.

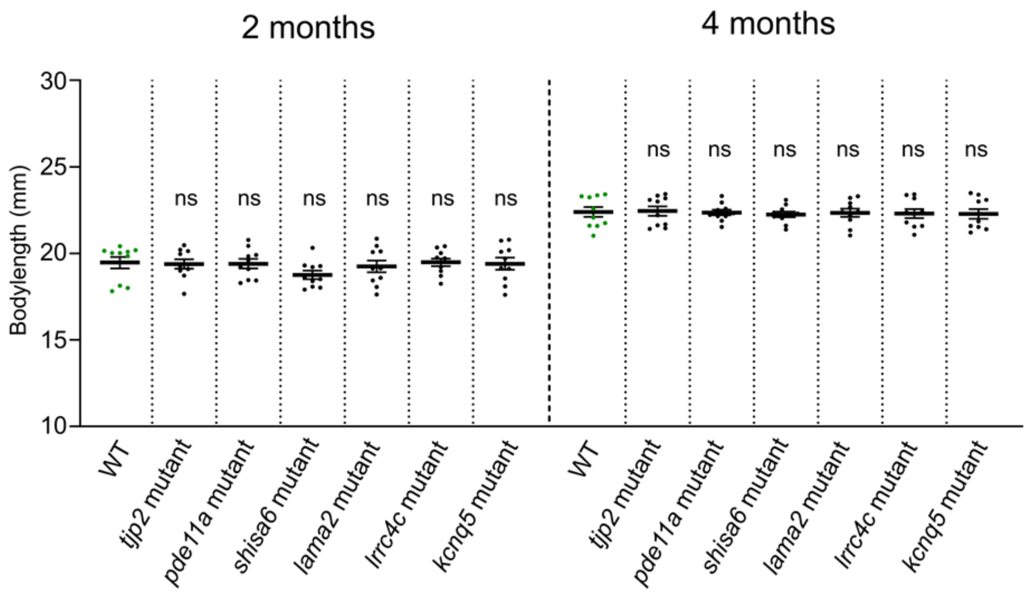

Body length measurements of the WT and mutant fish used in this study. Body length was measured from the tip of the head to the proximal end of the caudal fin. During spectral-domain optical coherence tomography, all mutant lines were compared to size-matched WT controls. Sample size: n=10 fish for n=20 eyes. Error bars: SEM. Significance: ns=not significant. WT: wild-type, *tjp2* mutant: *tjp2a*^{b1367}*tjp2b*^{b1368}, *pde11a* mutant: *pde11a*^{re13}*pde11a-like*^{re14}, *shisa6* mutant: *shisa6*^{re15}, *lama2* mutant: *lama2*^{re16}, *lrrc4c* mutant: *lrrc4ca*^{re17}*lrrc4cb*^{re18}, *kcnq5* mutant: *kcnq5a*^{re19}*kcnq5b*^{re20}.

Supplementary Figure S2. Circularity and curvature of WT and tjp2.

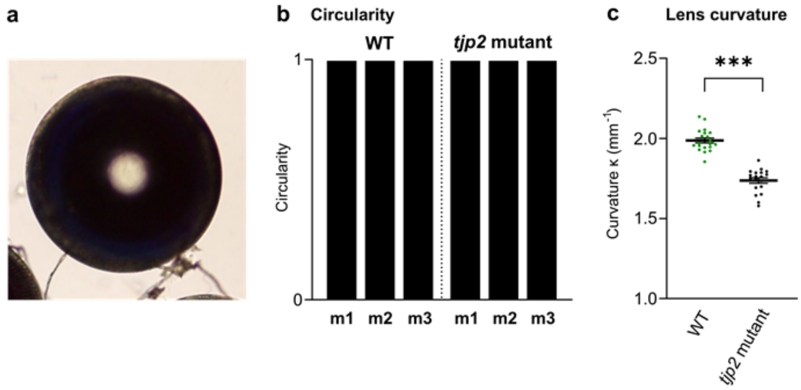

a Example image showing a circle on the border of the great circle of the spherical zebrafish lens. **b** Enucleated lenses were photographed from 3 positions. The circularity of the great circles (mid planes) of all lenses were equal to 1. Sample size: n=10 fish; n=20 lenses. **c** Curvature analysis of SD-OCT data shows that the curvature (K) is increased for the larger *tjp2* mutant lens. Sample size: n=10 fish; n=20 lenses. Error bars: SEM. ***$p < 0.001$. WT: wild-type, *tjp2* mutant: *tjp2a*^{b1367}*tjp2b*^{b13688}, M: measurement #.

Supplementary Figure S3. Full-length gels included in Fig. 2

βActin (437 bp)

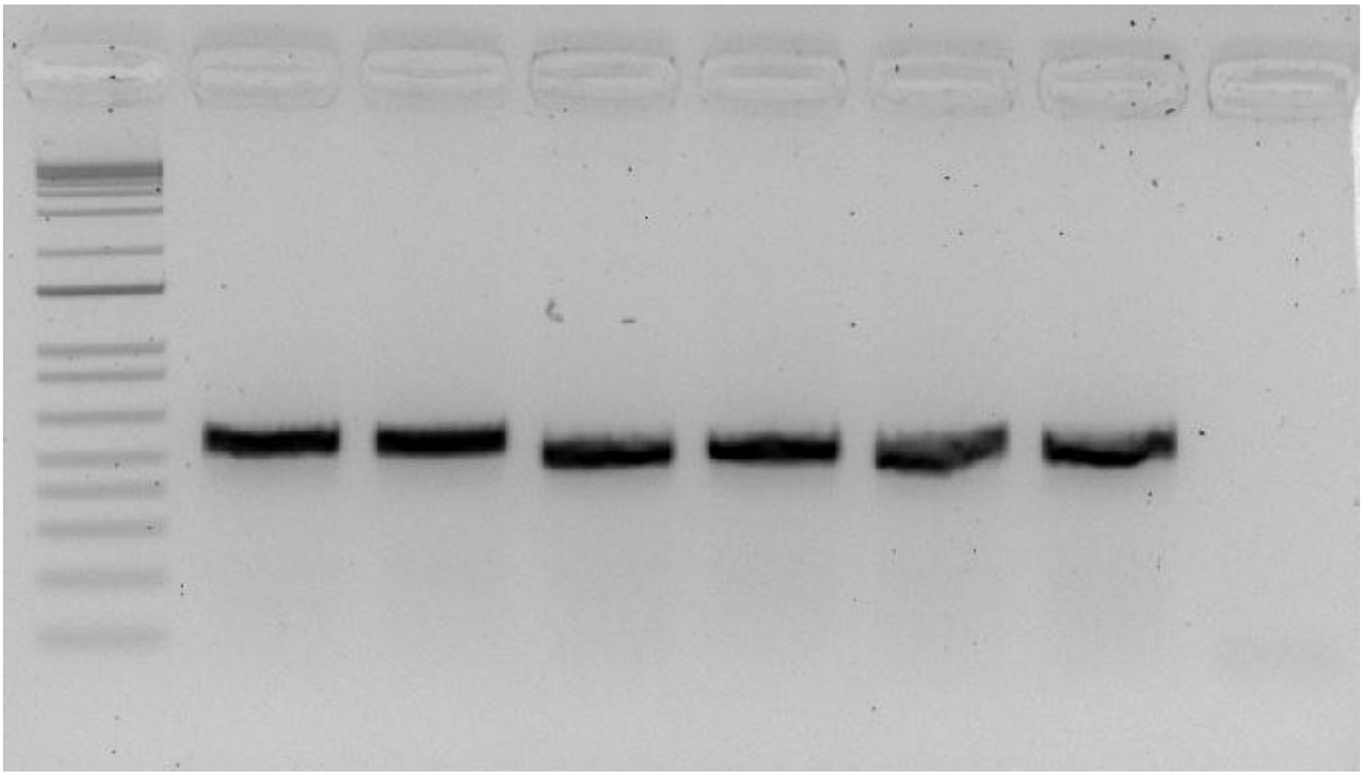

tjp2a (914 bp)

M 4d 1m 2m 3m 6m 1y W

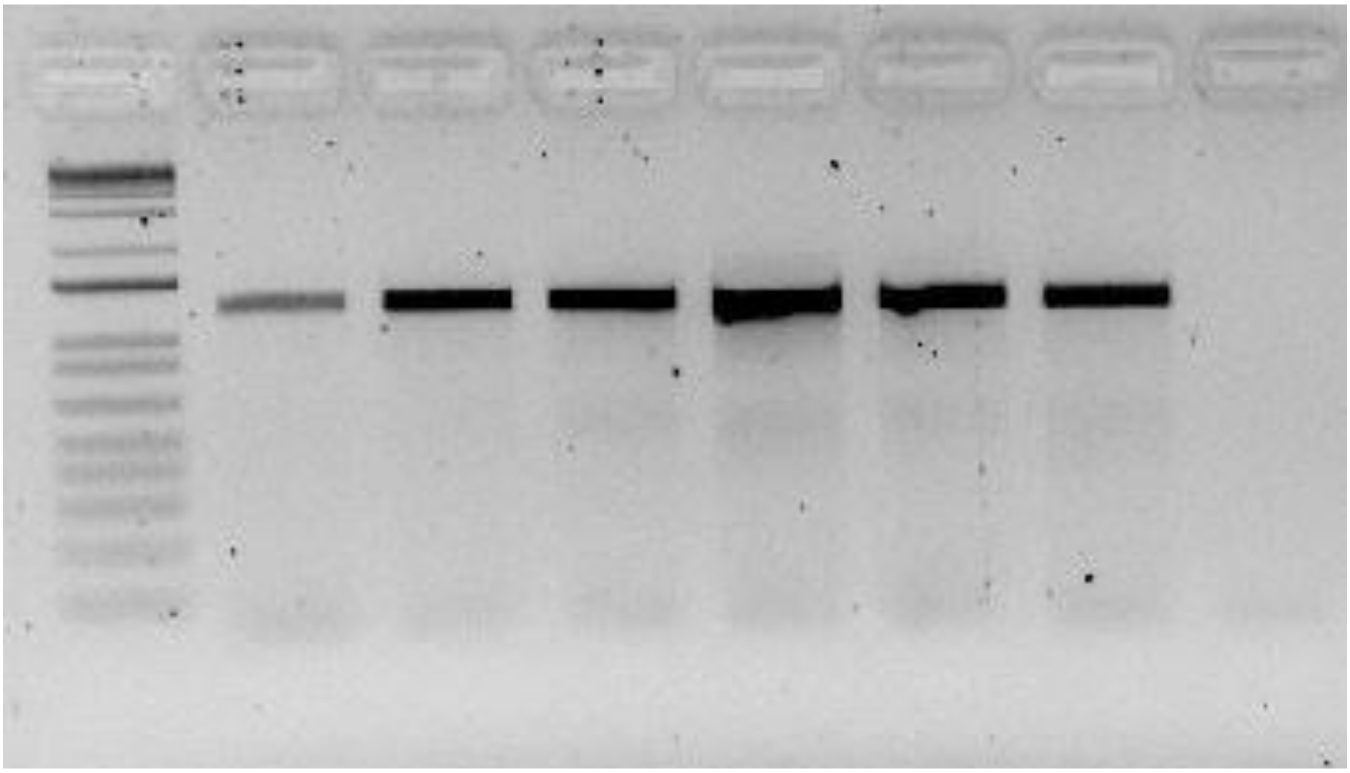

tjp2b (718 bp)

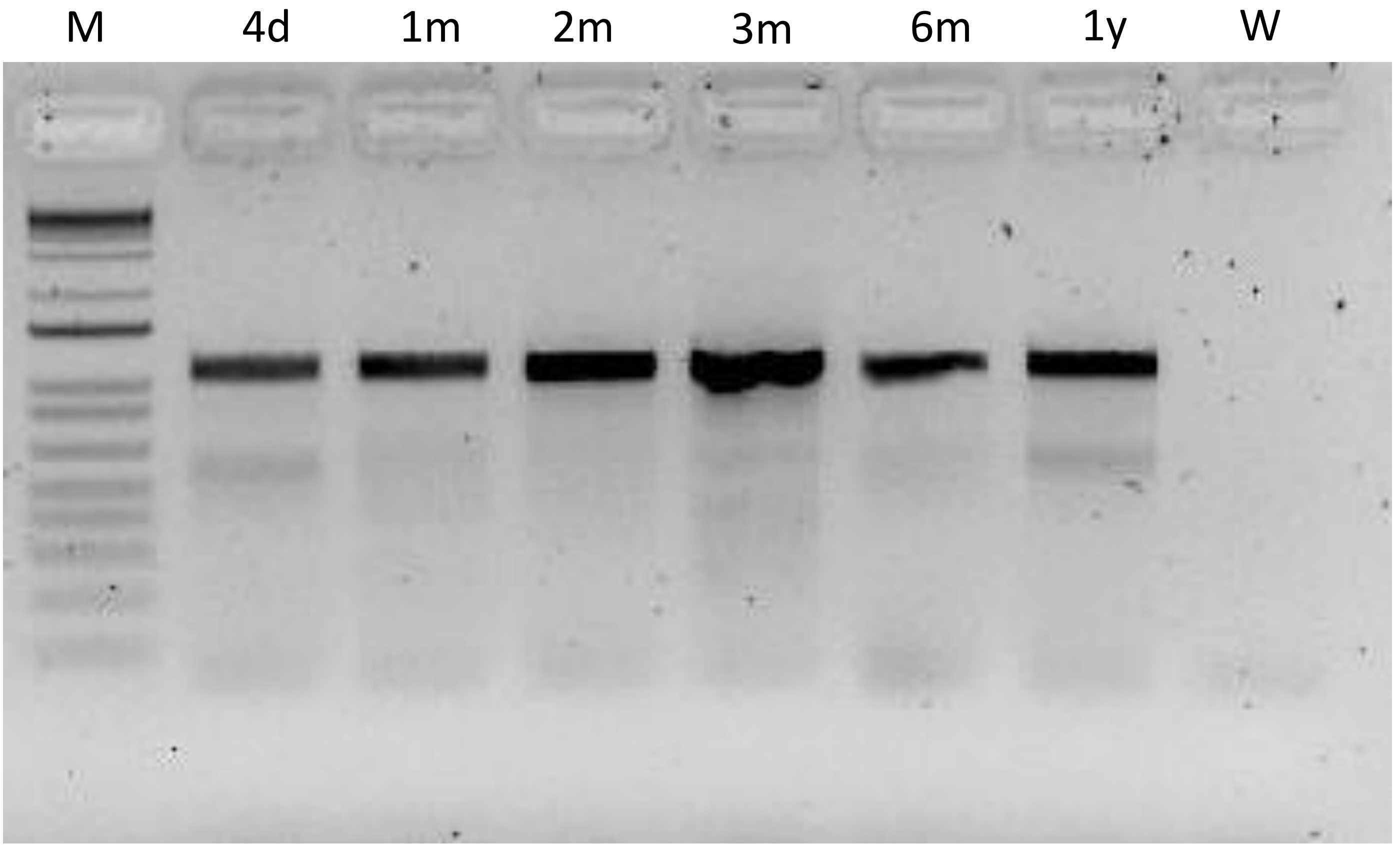

pde11a (896 bp)

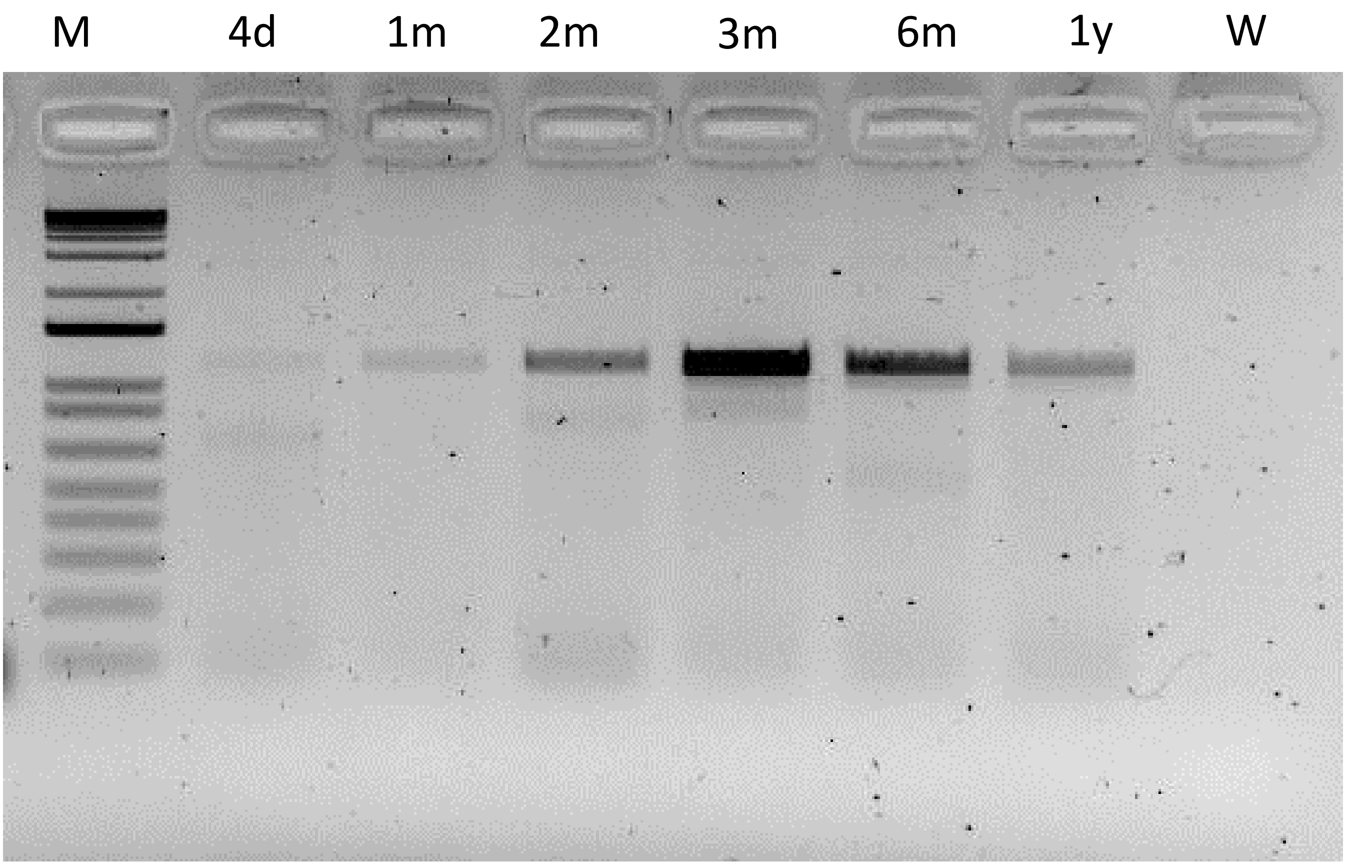

pde11a-l (716)

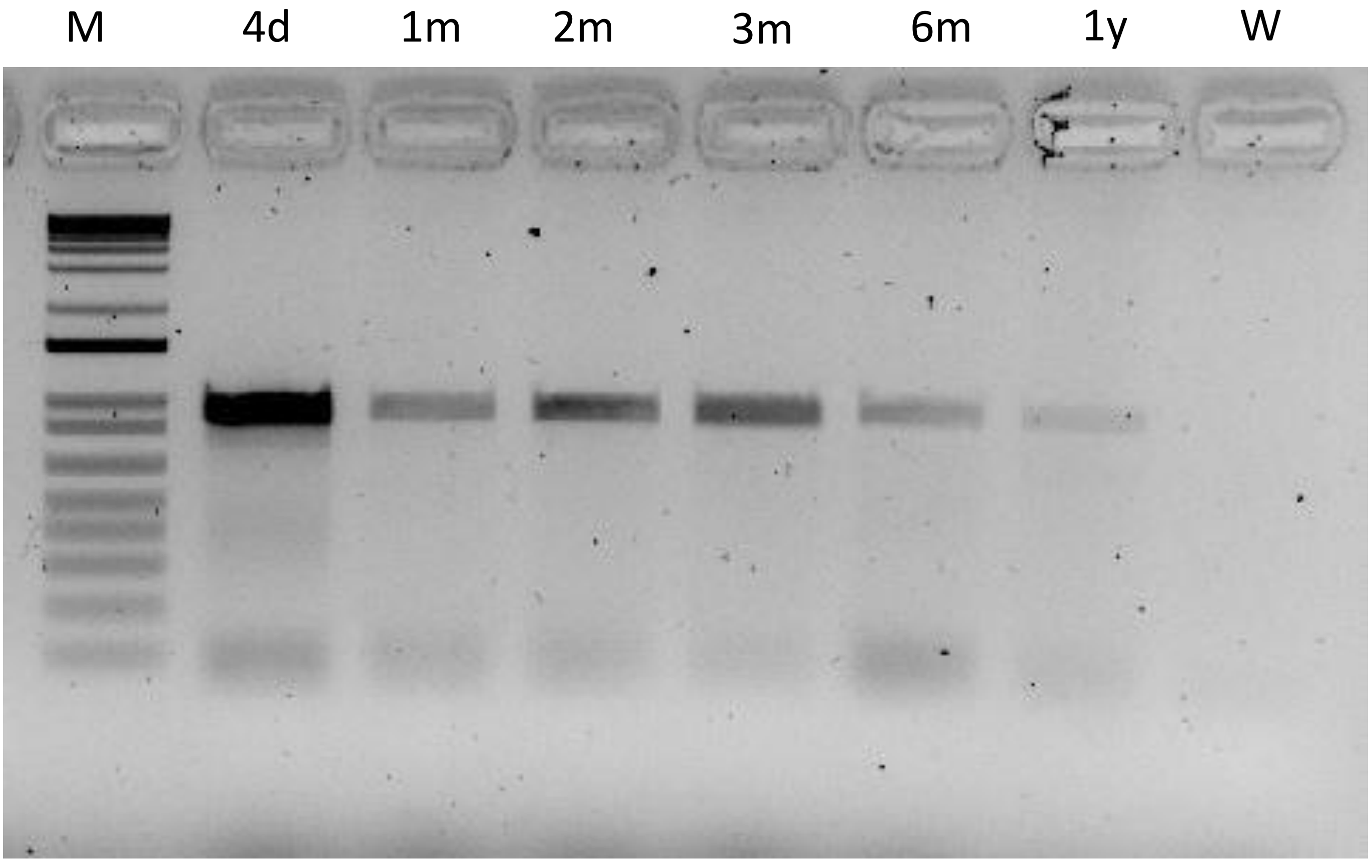

shisa6 (699 bp)

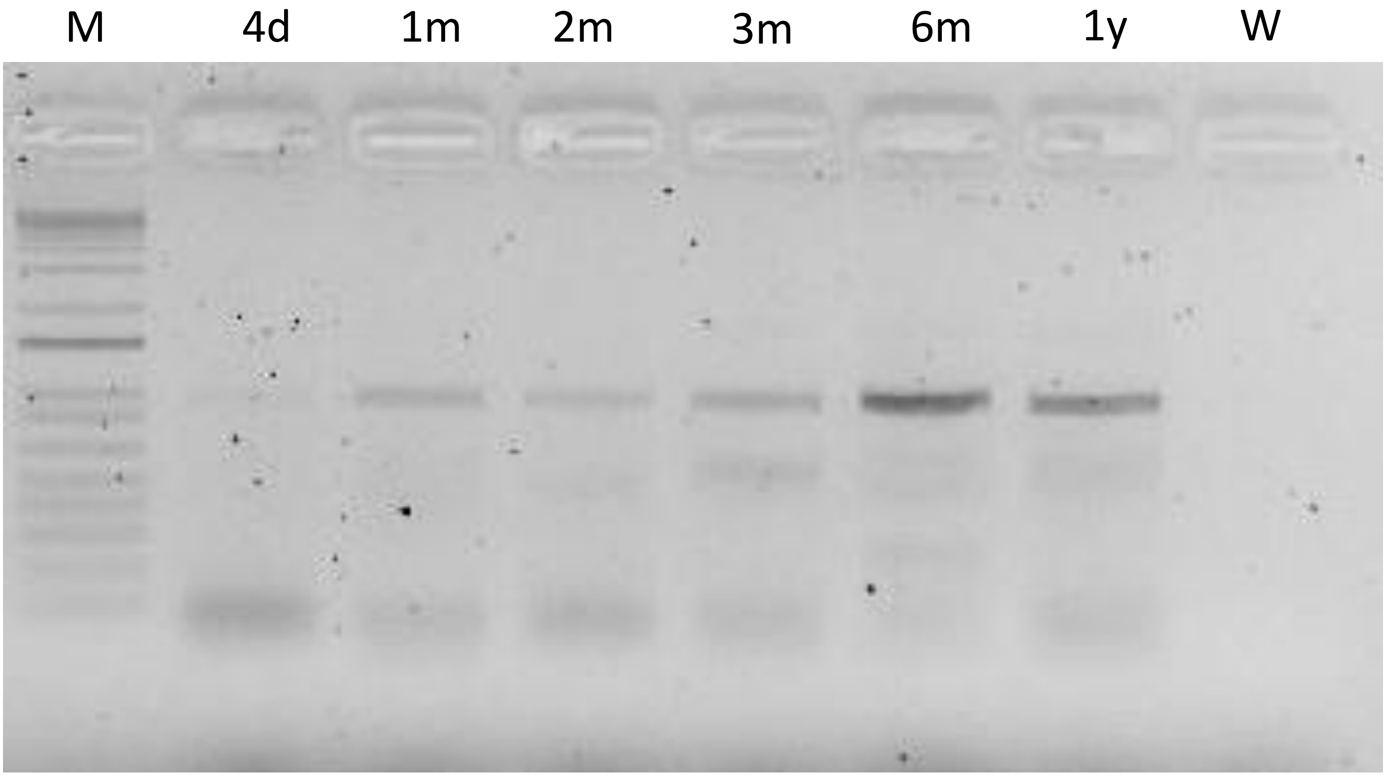

lama2 (893 bp)

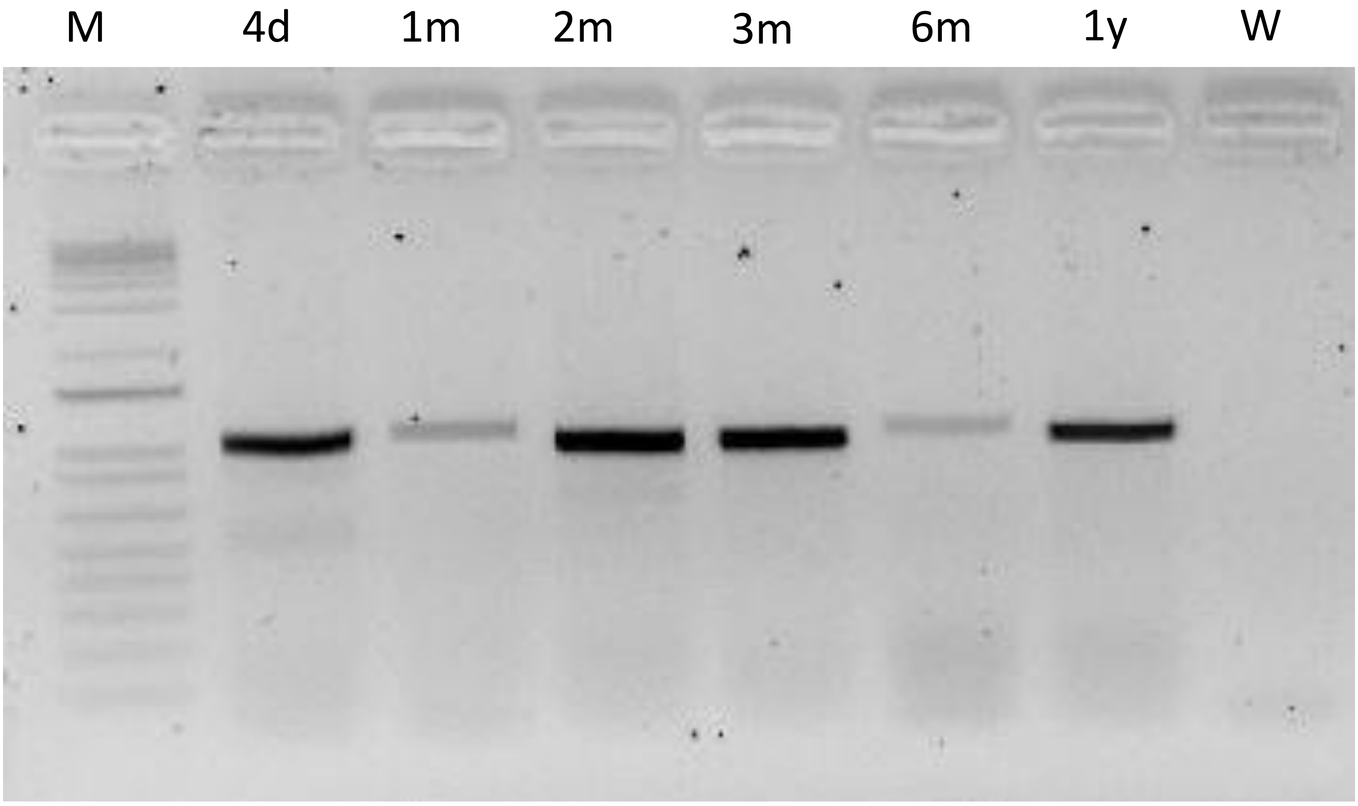

lrrc4ca (817 bp)

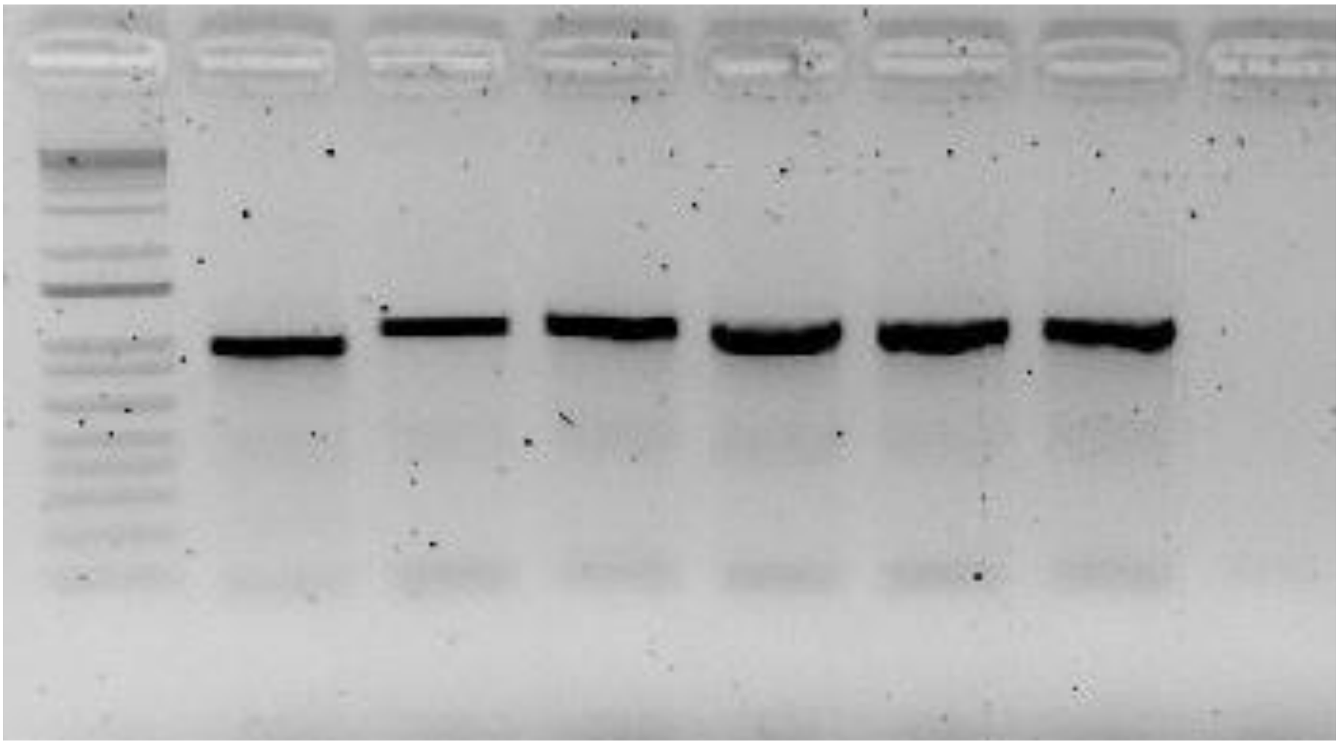

lrrc4cb (845 bp)

M 4d 1m 2m 3m 6m 1y W

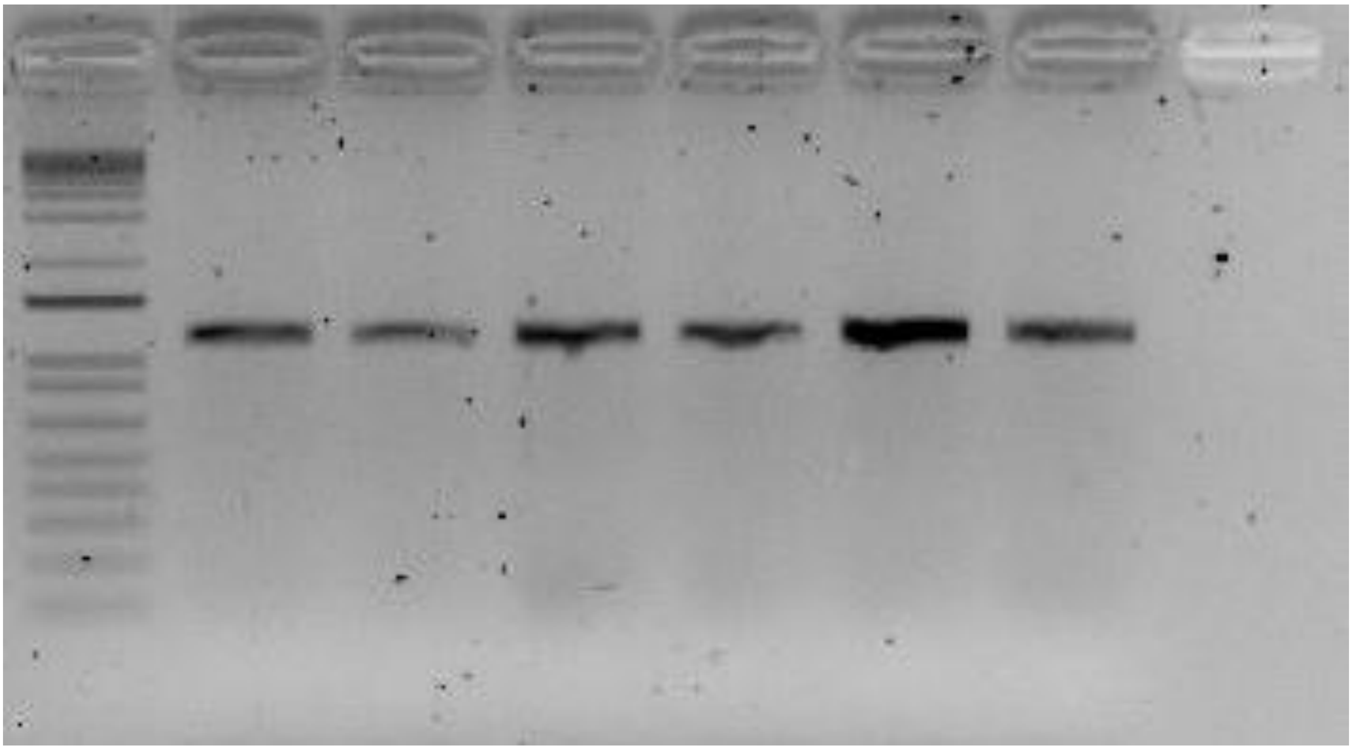

kcnq5a (906 bp)

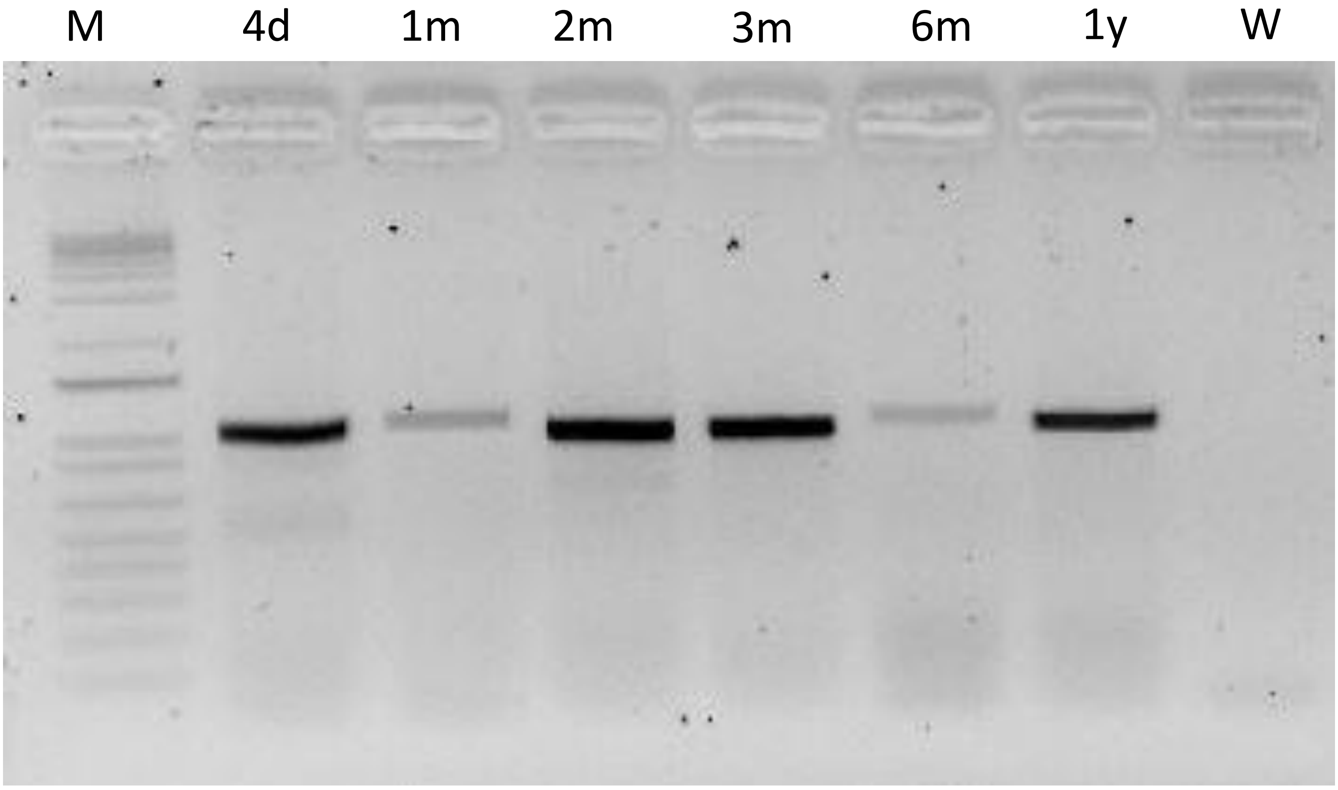

***kcnq5b* (755 bp)**

M 4d 1m 2m 3m 6m 1y

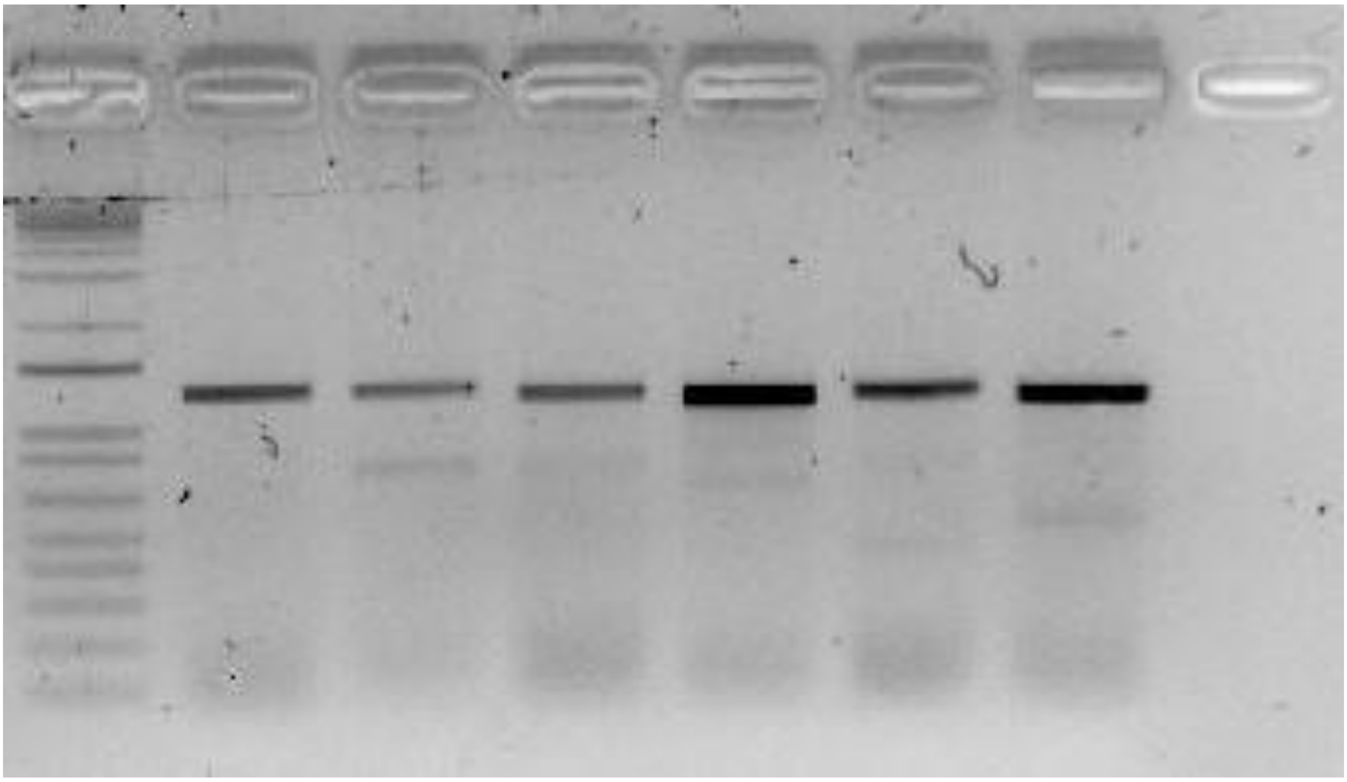

gnb3a (686 bp)

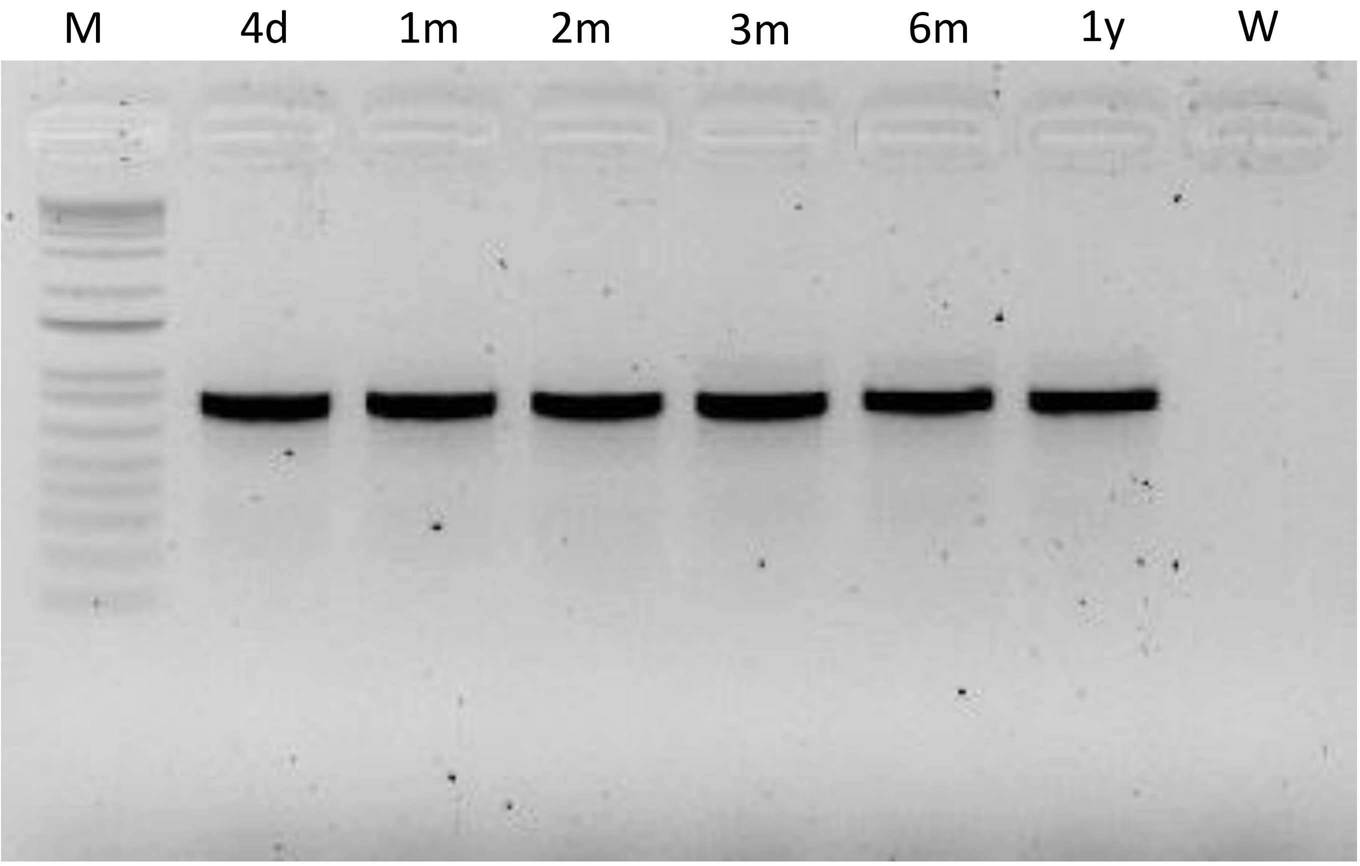

gnb3b (486 bp)

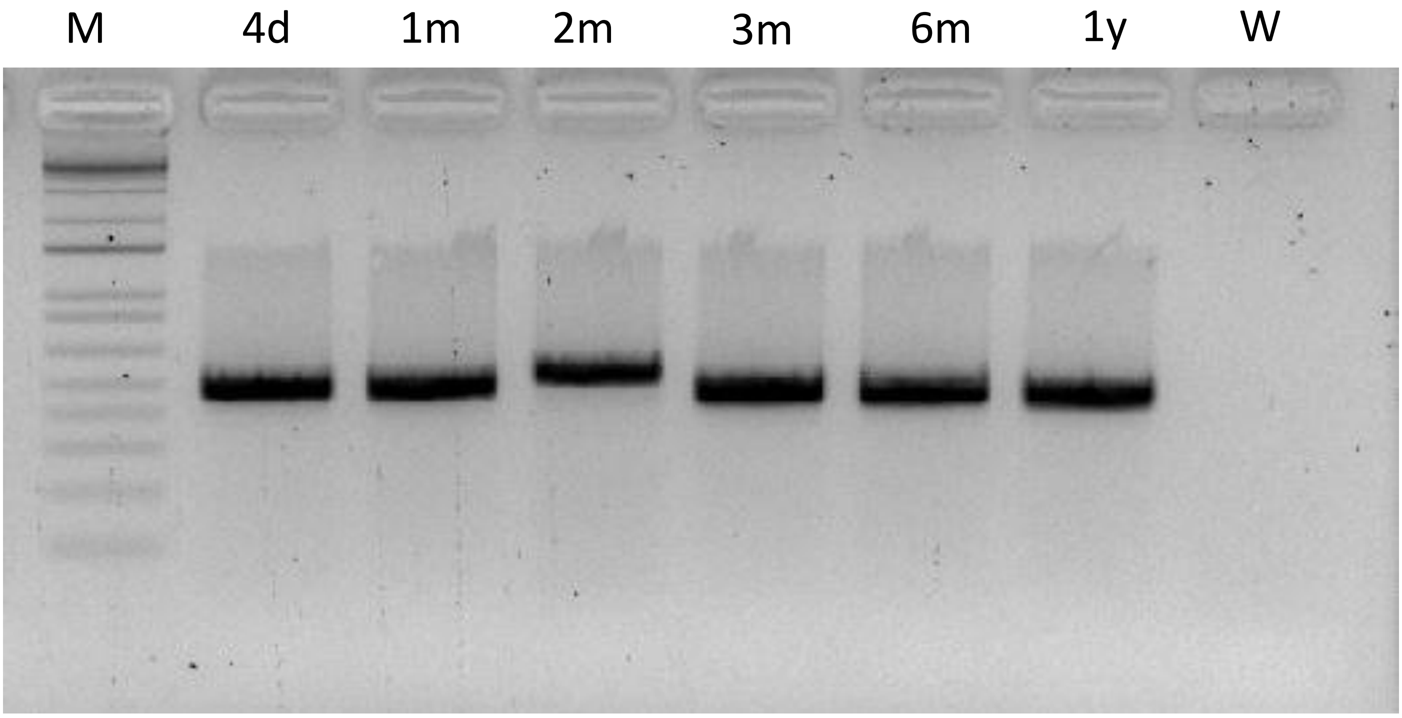

rbfox1 (584 bp)

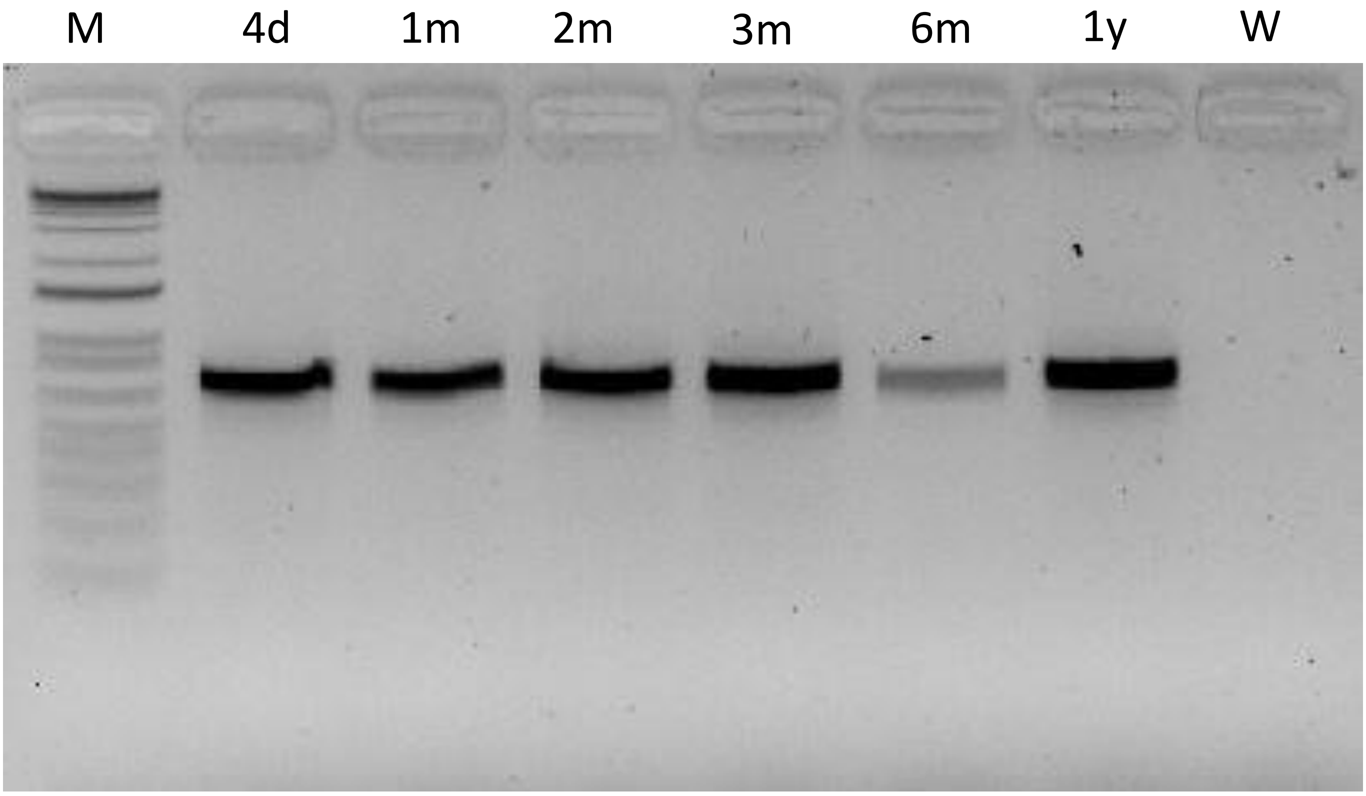

rbfox-1l (512 bp)

M 4d 1m 2m 3m 6m 1y W

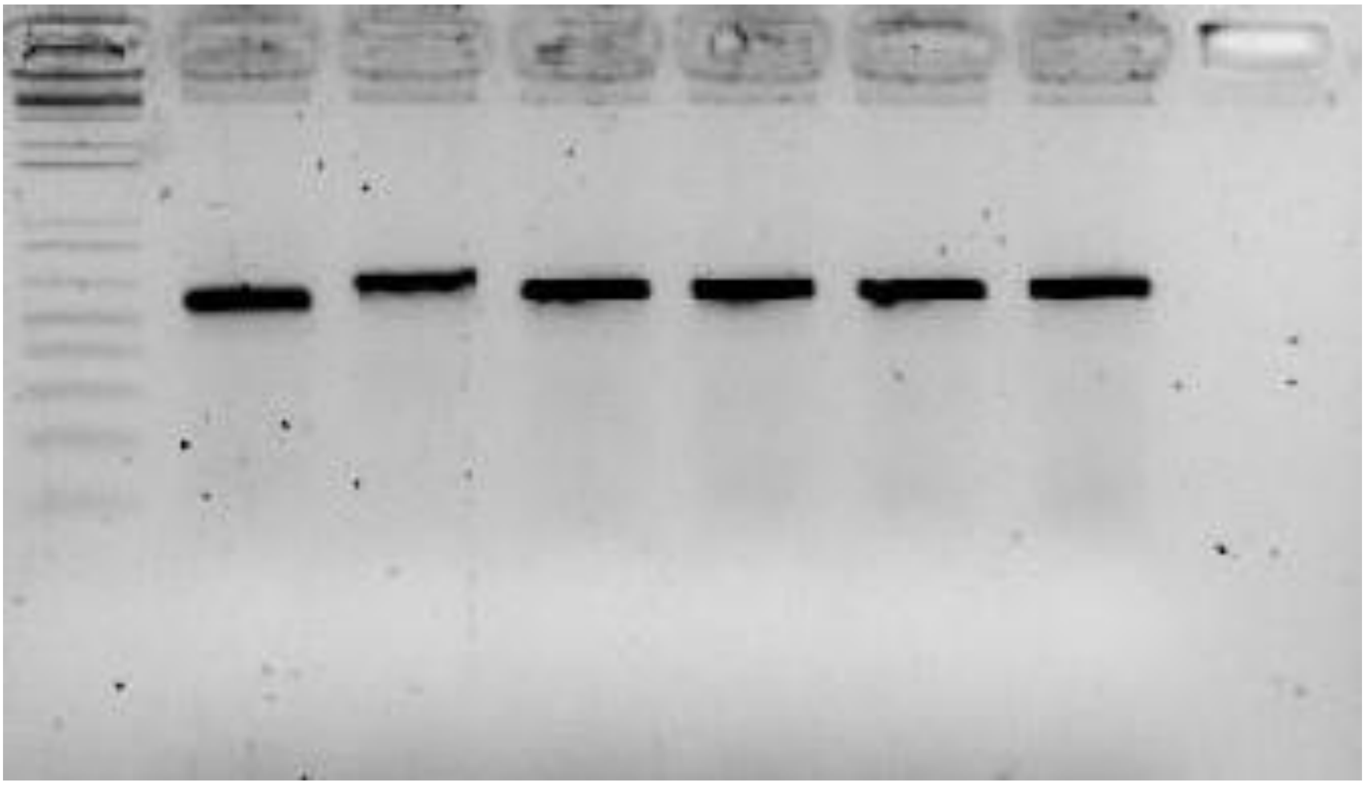

gria4a (583 & 464 bp)

M 4d 1m 2m 3m 6m 1y W

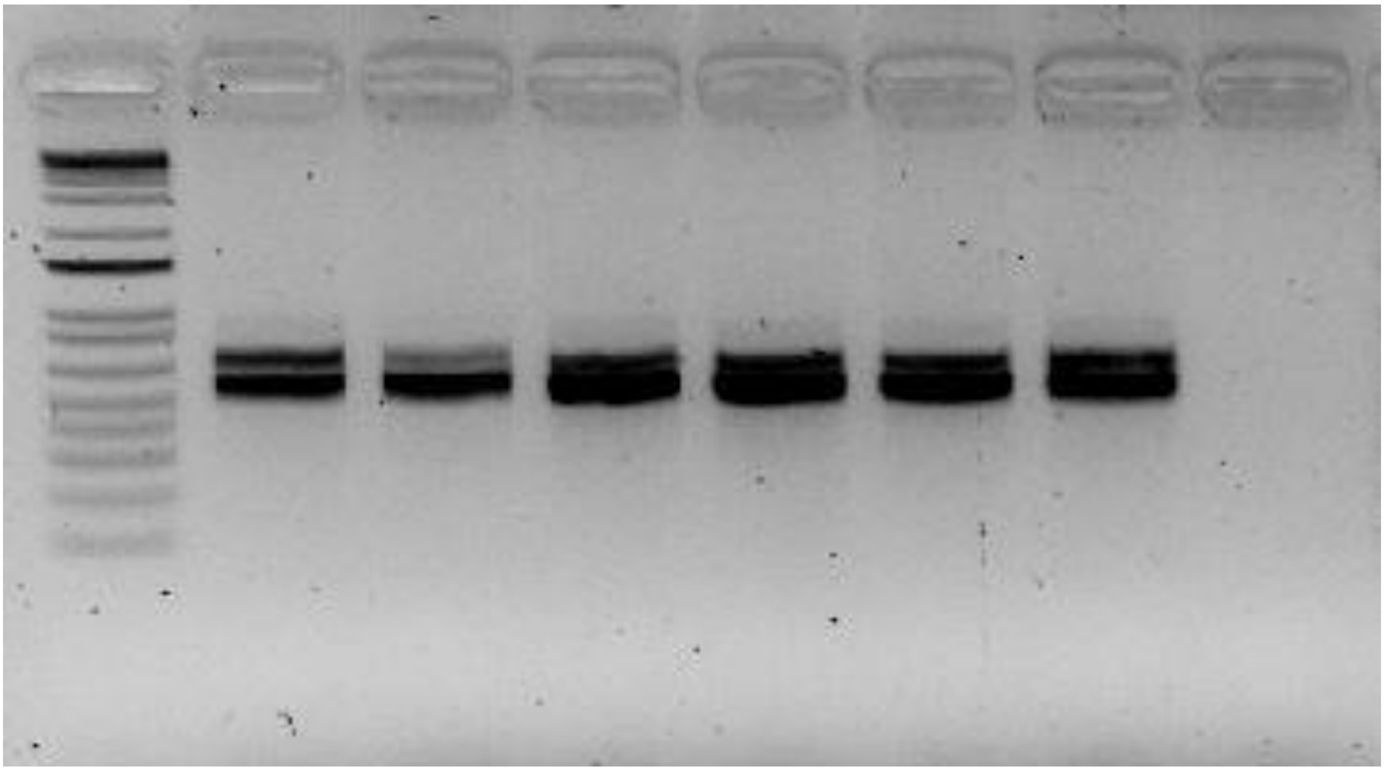

gria4a (583 & 464 bp)

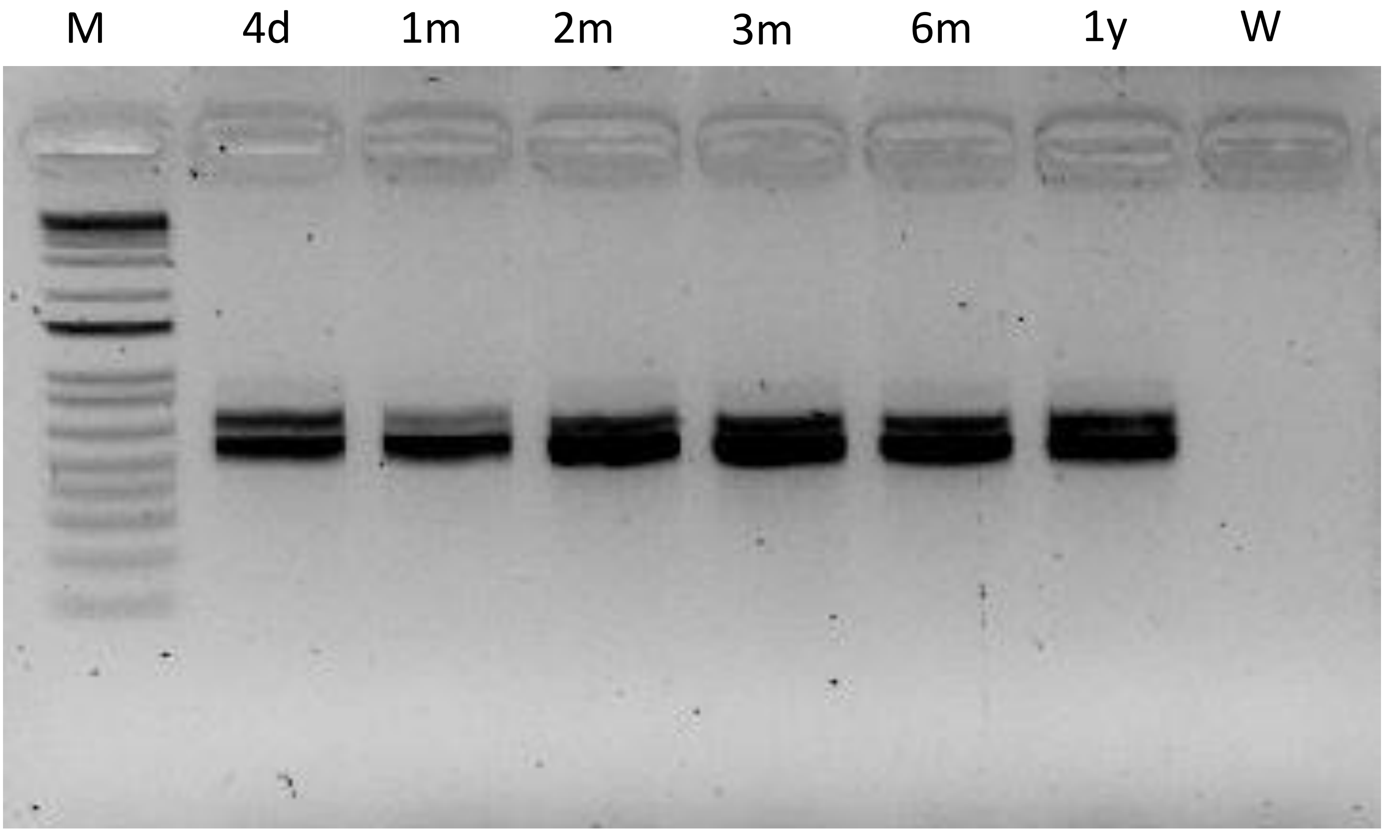

Supplement: Supplementary file 6 — Supplementary Information 6. [file 41598_2023_28944_MOESM6_ESM.pdf]
